# An Integrated Comprehensive Peptidomics and In Silico Analysis of Bioactive Peptide-Rich Milk Fermented by Three Autochthonous Cocci Strains

**DOI:** 10.3390/ijms25042431

**Published:** 2024-02-19

**Authors:** Martina Banić, Katarina Butorac, Nina Čuljak, Ana Butorac, Jasna Novak, Andreja Leboš Pavunc, Anamarija Rušanac, Željka Stanečić, Marija Lovrić, Jagoda Šušković, Blaženka Kos

**Affiliations:** 1 Laboratory for Antibiotic, Enzyme, Probiotic and Starter Culture Technologies, Faculty of Food Technology and Biotechnology, University of Zagreb, Pierottijeva 6, 10000 Zagreb, Croatia; mmarijanovic@pbf.unizg.hr (M.B.); katarina.butorac@pbf.unizg.hr (K.B.); nina.culjak@pbf.unizg.hr (N.Č.); jasna.novak@pbf.unizg.hr (J.N.); andreja.lebos.pavunc@pbf.unizg.hr (A.L.P.); arusanac@pbf.hr (A.R.); jsusko@pbf.hr (J.Š.); 2 BICRO Biocentre Ltd., Borongajska cesta 83H, 10000 Zagreb, Croatia; ana.butorac@biocentre.hr (A.B.); zeljka.stanecic@biocentre.hr (Ž.S.); marija.lovric@biocentre.hr (M.L.)

**Keywords:** bioactive peptides, peptidomics, *Lactococcus*, *Enterococcus*, lyophilisation, microbial fermentation

## Abstract

Bioactive peptides (BPs) are molecules of paramount importance with great potential for the development of functional foods, nutraceuticals or therapeutics for the prevention or treatment of various diseases. A functional BP-rich dairy product was produced by lyophilisation of bovine milk fermented by the autochthonous strains *Lactococcus lactis* subsp. *lactis* ZGBP5-51, *Enterococcus faecium* ZGBP5-52 and *Enterococcus faecalis* ZGBP5-53 isolated from the same artisanal fresh cheese. The efficiency of the proteolytic system of the implemented strains in the production of BPs was confirmed by a combined high-throughput mass spectrometry (MS)-based peptidome profiling and an in silico approach. First, peptides released by microbial fermentation were identified via a non-targeted peptide analysis (NTA) comprising reversed-phase nano-liquid chromatography (RP nano-LC) coupled with matrix-assisted laser desorption/ionisation-time-of-flight/time-of-flight (MALDI-TOF/TOF) MS, and then quantified by targeted peptide analysis (TA) involving RP ultrahigh-performance LC (RP-UHPLC) coupled with triple-quadrupole MS (QQQ-MS). A combined database and literature search revealed that 10 of the 25 peptides identified in this work have bioactive properties described in the literature. Finally, by combining the output of MS-based peptidome profiling with in silico bioactivity prediction tools, three peptides (^75^QFLPYPYYAKPA^86^, ^40^VAPFPEVFGK^49^, ^117^ARHPHPHLSF^126^), whose bioactive properties have not been previously reported in the literature, were identified as potential BP candidates.

## 1. Introduction

Proteins are among the most important essential macronutrients that not only provide nutrients and amino acids essential for growth and development but also contain specific fragments known as bioactive peptides (BPs), which have beneficial physiological effects on human health. From a therapeutic perspective, BPs have numerous advantages over synthetic chemical compounds commonly used as drugs, as they are more stable, more easily absorbed and act specifically on the target tissue at low concentrations without toxic effects [1,2,3]. Therefore, BPs have great potential to be used as functional foods, nutraceuticals or therapeutics for the prevention or treatment of various chronic diseases. Numerous functions attributed to food-derived BPs encompass antimicrobial, antioxidant, antiamnestic, antimutagenic, anticarcinogenic, antihypertensive, antihaemolytic, anti-inflammatory, antithrombotic, immunomodulatory, cytomodulatory, osteogenic, appetite suppressant, mineral-binding or opioid activities, depending on their structure [4,5,6]. Although some BPs occur in a bare format, many are encrypted in a latent form within the intact structure of parent protein molecules and are activated by extraction [1,5]. However, most methods for releasing BPs are laborious, time-consuming and expensive on an industrial scale. The microbial fermentation of milk proteins using lactic acid bacteria (LAB) has emerged as an attractive approach for the production of BP-enriched functional foods due to its cost-effectiveness, environmental friendliness and the positive nutritional image associated with fermented dairy products [7,8]. LAB are not only the most important group of probiotic microorganisms but also the most commonly used starter cultures in the production of fermented foods, especially fermented dairy products, which are known to be the largest source of food-derived BPs [9]. BP synthesis through the proteolytic activity of LAB is attractive to the dairy industry, as the bioprocess contributes to the quality of the final product without the need for additional technological procedures and costs [10]. Both lactococci and enterococci have been featured in the dairy industry for decades as autochthonous non-starter and starter LAB, due to their interesting biotechnological properties, such as the production of organic acids, exopolysaccharides, BPs and bacteriocins [11,12]. In our earlier study [13], the autochthonous LAB strain *Lactococcus* (*Lc.*) *lactis* subsp. *lactis* ZGBP5-51, isolated from Croatian artisanal fresh cheese, showed a fast milk-coagulating (Fmc^+^) phenotype after 16 h of cultivation, indicating the high proteolytic potency of the strain, as most lactococci isolated from fermented dairy products show auxotrophy for several amino acids, and their growth in a protein-rich medium depends on the expression of a complex proteolytic system for the degradation of casein [14]. Like lactococci, enterococci are also auxotrophic for several amino acids and therefore require an efficient proteolytic system to release essential amino acids from peptides in order to grow in dairy matrices [15]. Previously, we showed that enterococci (*Enterococcus* (*E.*) *faecalis*, *E. faecium* and *E. durans*) accounted for almost 30% of all strains isolated from artisanal cheese [16]. As commensals, enterococci colonise the digestive system and are involved in the modulation of the immune system in humans and animals [17]. They are also used in the production of a variety of fermented and non-fermented foods, as well as for food preservation, as they produce bacteriocins, and to improve the nutritional value of food and feed [18]. However, concerns over the safety of *Enterococcus* strains as probiotics have been raised due to their inclination to acquire various virulence factors and resistance to many commonly used antibiotics, especially vancomycin, entailing their possible implication in the development of nosocomial infections [17]. Therefore, the absence of transferable antibiotic resistance genes and/or potential virulence determinants should be confirmed for any new potential probiotic strain proposed for the food or pharmaceutical industry [19]. The strains *E. faecium* SF68^®^ and *E. faecalis* Symbioflor1^®^, whose safety is supported by surveillance and well-documented scientific evidence for human and animal applications, are used for restoring the balance of the host intestinal microflora and for the auxiliary treatment of diarrhoea [20,21]. Due to their generally low acidification capacity, enterococci are often not regarded as primary starter cultures in the dairy industry. However, due to their specific biochemical properties such as proteolysis, lipolysis, esterase activity and citrate metabolism, they are often selected as adjunct or protective cultures and thus contribute to the typical taste, texture, flavour and rheological properties of dairy foods [22,23]. For instance, an increased content of water-soluble nitrogen and an increased proteolytic index were observed when *E. faecalis* strains were used as adjunct starters in cheese [24,25]. Therefore, the autochthonous strains *E. faecium* ZGBP5-52 and *E. faecalis* ZGBP5-53, isolated from the same artisanal fresh cheese, were used as adjunct starters to strain *Lc. lactis* subsp. *lactis* ZGBP5-51 in the fermentation of fresh bovine milk to produce the functional BP-rich lyophilised dairy product. The aim of this work was to identify and quantify BPs in the milk fermented by the autochthonous strains that could potentially be used as nutraceutical ingredients in functional foods and/or as pharmaceuticals due to their nutritional value and health-promoting properties, and to utilise available in silico bioactivity prediction tools to identify potential new BP candidates whose bioactive properties have not yet been described in the literature. Therefore, the efficiency of the proteolytic system of the selected strains, ZGBP5-51, ZGBP5-52 and ZGBP5-53, in the production of BPs in the casein-rich matrix was evaluated by a combined high-throughput mass spectrometry (MS)-based peptidomic profiling of the produced lyophilised functional fermented dairy product and an in silico approach. The BPs released by the microbial fermentation were first identified by non-targeted analysis (NTA) of peptides and then quantified by targeted peptide analysis (TA). Finally, the output of LC-MS-based peptidome profiling was combined with amino acid composition-based in silico prediction of bioactivity to identify a number of potential new BP candidates.

## 2. Results and Discussion

Beyond their basic nutritional value, foods are now recognised as a source of biologically active compounds that exert targeted physiological effects in the body, thus improving human health and the general well-being of the organism. So-called functional foods represent a lucrative market with a growth rate outpacing that of the overall food industry. Business Communications Company (BCC) research estimates the global functional food and beverage market at USD 216.4 billion in 2022 and forecasts a compound annual growth rate of 8.4% from 2022 to 2027, indicating tremendous expansion [26]. Scientific research on BPs has recently proliferated, mainly due to their prodigious potential in the prevention and treatment of various diseases. The BIOPEP-UWM^TM^ database currently contains information on 4746 BPs with various biological activities. In this work, the autochthonous strains *Lc. lactis* subsp. *lactis* ZGBP5-51, *E. faecium* ZGBP5-52 and *E. faecalis* ZGBP5-53, isolated from the same artisanal fresh cheese, were used for the production of the functional fermented BP-rich lyophilised dairy product.

### 2.1. Whole-Genome Sequencing (WGS) and Genome Annotation of Bacterial Strains

**Figure 1:**
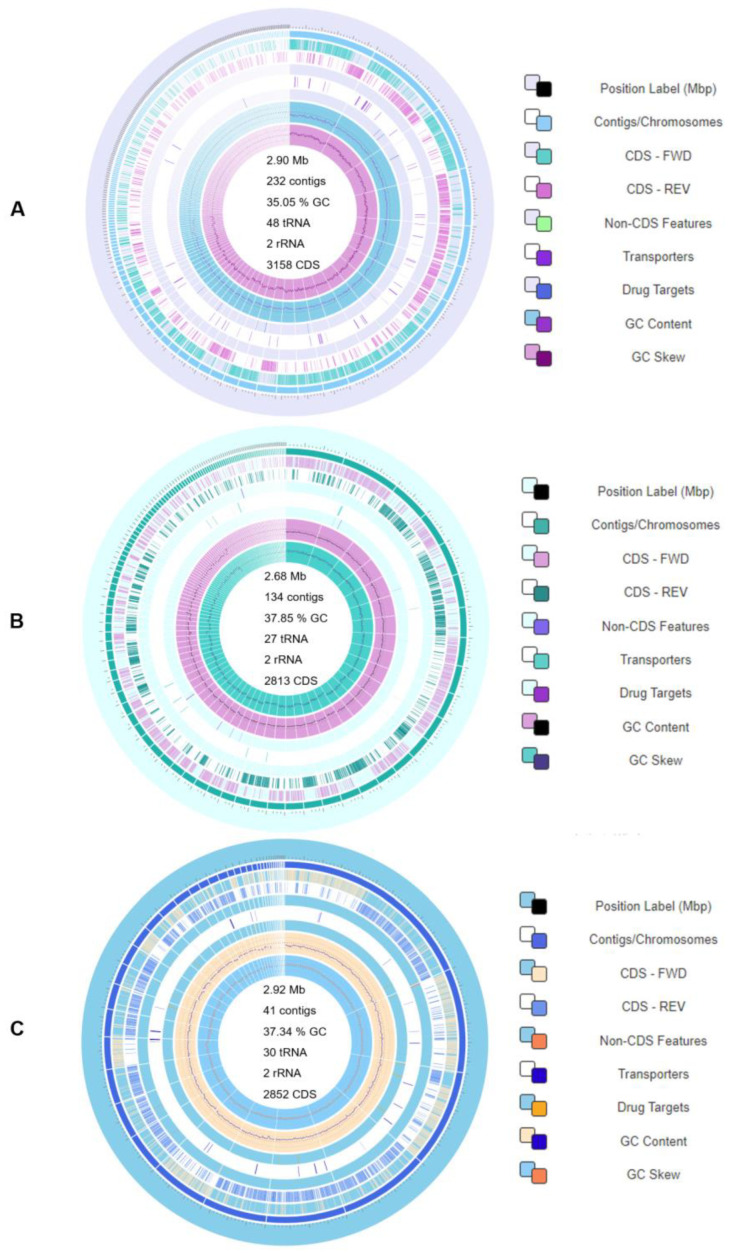
Circular genome maps of (**A**) *Lc. lactis* subsp. *lactis* ZGBP5-51, (**B**) *E. faecium* ZGBP5-52 and (**C**) *E. faecalis* ZGBP5-53. From the inner to the outer rings: guanine and cytosine (GC) skew, GC content, genes for drug targets, genes for transporters, non-coding features, coding sequence on the reverse strand (CDS-REV), CDS on the forward strand (CDS-FWD), position and order of assembled contigs and reference position in the genome (×1 Mbp).

Circular genome maps of the annotated genomes of *Lc. lactis* subsp. *lactis* ZGBP5-51, *E. faecium* ZGBP5-52 and *E. faecalis* ZGBP5-53 were generated using the Circular Viewer functionality built within the Bacterial and Viral Bioinformatics Resource Center (BV-BRC) system (Figure 1). Strain *Lc. lactis* subsp. *lactis* ZGBP5-51 contains 2,901,648 nucleotides (nt) with a total guanine and cytosine (GC) content of 35.05% in 232 contigs (Figure 1A). The genome of strain *E. faecium* ZGBP5-52 comprises 2,679,909 nt, distributed in 134 contigs, with a GC content of 37.85% (Figure 1B), while strain *E. faecalis* ZGBP5-53 has a larger genome with 2,917,252 nt, distributed in only 41 contigs, but a similar GC content of 37.34% (Figure 1C). The diversity of the proteolytic systems of strains ZGBP5-51, ZGBP5-52 and ZGBP5-53 was demonstrated by in silico analysis of the genes involved in their proteolytic activity, using the BV-BRC system. The analysis showed that the genomes of the implemented strains harbour a large number of genes involved in proteolytic activity. Namely, the genome of strain *Lc. lactis* subsp. *lactis* ZGBP5-51 contains at least 39 genes involved in proteolytic activity, while the genomes of strains *E. faecium* ZGBP5-52 and *E. faecalis* ZGBP5-53 contain at least 45 and 46 proteolytic genes, respectively (Supplementary Table S1). The annotated genomes were then analysed in silico, using the BV-BRC system for the possible presence of an antimicrobial resistance (AMR) phenotype and cytolysins A, B and M (*cylA*, *cylB* and *cylM*); hyaluronidase (*hyl*); *faecalis* endocarditis antigen (*efaA*); pheromones (*cpd*, *cob* and *ccf*); and extracellular superoxide, which are the most important virulence determinants of enterococci [27]. Since vancomycin-resistant enterococci (VRE) are one of the major causes of nosocomial infections [17], it was crucial to verify the absence of glycopeptide resistance in enterococci mediated by eight (*vanA*, *vanB*, *vanD*, *vanE*, *vanG*, *vanL*, *vanM* and *vanN*) mobile gene clusters [28]. The absence of both chromosomally integrated vancomycin resistance genes and those located on mobile genetic elements was confirmed for *E. faecium* ZGBP5-52 and *E. faecalis* ZGBP5-53. The absence of a general AMR phenotype predicted by the BV-BRC system was also confirmed for all three implemented strains. The absence of an AMR phenotype and the abovementioned virulence determinants in the genomes of all three implemented strains was confirmed, supporting the hypothesis of their safe use in the production of BPs.

### 2.2. Preparation of the Functional Fermented BP-Rich Lyophilised Dairy Product

**Figure 2:**
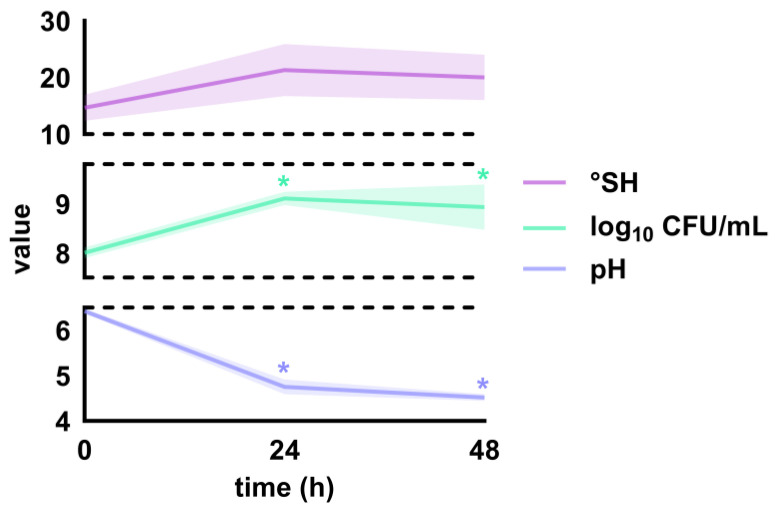
Fermentation parameters, i.e., the degree of acidity (°SH), pH and cell viability of cocci grown on M17 agar, determined before and after 24 and 48 h of fermentation. The shaded coloured areas around the lines mark error bands, i.e., the dynamics of the change in the determined values of the fermentation parameters. The asterisks (*) indicate values with a significant difference (*p* < 0.05) compared to the value of each fermentation parameter determined before fermentation (at 0 h).

*Lc. lactis* subsp. *lactis* ZGBP5-51, *E. faecium* ZGBP5-52 and *E. faecalis* ZGBP5-53 were used for the production of the functional lyophilised fermented dairy product. During the 48-h cultivation of the three selected strains in milk, certain fermentation parameters were monitored. First, the cell viability of the applied strains accompanied with other cocci naturally present in the milk was determined before and after 24 and 48 h of fermentation. As shown in Figure 2, a significant (*p* = 0.77 × 10^−2^) increase in bacterial counts of 1.10 ± 0.18 log_10_ units was observed after 24 h of fermentation. Such an increase was predictable as the bacteria entered the log phase. After 48 h of fermentation, the viable cell count decreased negligibly (*p* = 0.75) by 0.17 ± 0.48 log_10_ units as the bacteria passed through the stationary phase and entered the death phase. Meanwhile, the degree of acidity (°SH) increased from 14.67 ± 2.39 to 21.33 ± 4.62 (*p* = 0.16) after 24 h in proportion to the increase in the number of viable cells, and then it decreased slightly to 20.00 ± 4.00 (*p* = 0.90) after 48 h, corresponding to the slight decrease in the number of viable cells. Contrariwise, the pH decreased significantly (*p* = 1.00 × 10^−8^) by 1.67 ± 0.07 units in the first 24 h of fermentation and additionally by 0.23 ± 0.08 units (*p* = 0.15 × 10^−2^) by the 48-h mark. Such a result was anticipated, as the growth of lactococci and enterococci produces acidic fermentation end products that accumulate in the extracellular environment, henceforth lowering its pH and creating unfavourable conditions for acid-sensitive microorganisms.

### 2.3. Peptidomic Profiling of the BP-Rich Lyophilised Dairy Product

The release of BPs during milk fermentation by the autochthonous strains ZGBP5-51, ZGBP5-52 and ZGBP5-53 was assessed by a combined high-throughput MS-based peptidomic profiling of the produced lyophilised fermented dairy product and an in silico approach. Peptidomics is a powerful multidisciplinary field that combines separation techniques such as modern LC and MS technologies, innovative bioinformatics and statistics for the qualitative and quantitative analysis of peptides present in a sample, which is nowadays crucial for delineating the properties of various types of food—inter alia, fermented foods—as well as for monitoring the abundance of already-known BPs in different matrices and for discovering novel BPs from the vast background of observed degradation products and inactive precursors [29,30,31].

**Figure 3:**
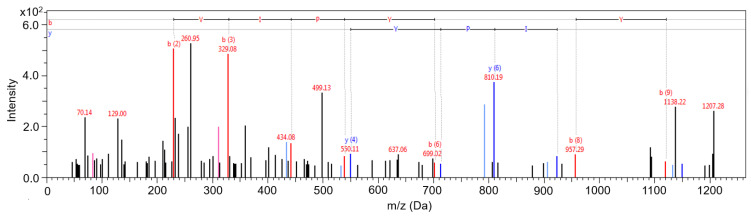
Representative MS/MS spectrum of peptide ^213^TKVIPYVRYL^222^ identified in the sample fermented with *Lactococcus* (*Lc.*) *lactis* subsp. *lactis* ZGBP5-51, *Enterococcus* (*E.*) *faecium* ZGBP5-52 and *E. faecalis* ZGBP5-53 after 24 h of fermentation. y ions are shown in blue and b ions in red. Unassigned black peaks could not be assigned to any sequence.

First, the BPs released in this study were identified in the lyophilised samples with NTA. Unfermented, uninoculated, pasteurised milk was used as a control because BPs can be released from milk proteins by pathways other than hydrolysis by microbial enzymes. These include milk secretion, storage, processing, enzymatic hydrolysis by indigenous and digestive enzymes, etc. [32,33] A total of 192 peptide fractions were chromatographically separated and purified with reversed-phase nano-liquid chromatography (RP nano-LC) and analysed by MS/MS with Autoflex speed matrix-assisted laser desorption/ionisation–time-of-flight/time-of-flight (MALDI-TOF/TOF). The MS/MS peptide spectra were searched against the SwissProt database, *Bos taurus* taxonomy, using ProteinScape 3.0, with the embedded search engine Mascot 2.5.0. A total of 22 peptides derived by the non-specific cleavage of various naturally occurring precursor proteins in milk by the action of the proteolytic system of the implemented strains were identified in the lyophilised fermented samples (Table 1). A representative assigned spectrum of peptide ^213^TKVIPYVRYL^222^ (*m*/*z* 1251.75 Da) identified after fragmentation by MS/MS is shown in Figure 3. The NTA did not detect any peptides in the control samples of unfermented, uninoculated, pasteurised milk. After 24 h of fermentation, merely 10 peptides were identified that had been released by non-specific cleavage of the precursor proteins α-s2-casein, β-casein and κ-casein, whereas after 48 h of fermentation, a total of 20 peptides were identified that were derived from α-s1-casein, β-casein and κ-casein, of which 13 peptides had not been revealed in the 24-h samples. An increase in the number of peptides released after 48 h of fermentation was anticipated, as the prolonged exposure to the proteolytic system of the implemented strains leads to more non-specifically cleaved milk proteins.

**Figure 4:**
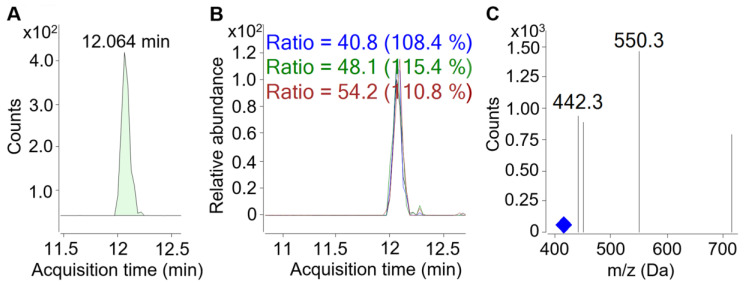
Representative MRM chromatograms of the peptide ^213^TKVIPYVRYL^222^ (*m*/*z* 417.9 Da), recorded in a sample of fermented milk. (**A**) MRM chromatogram of the fragment ion used for quantification (transition 417.9→550.3). (**B**) Superimposed MRM chromatograms of all fragment ions. (**C**) MS spectrum of all fragment ions. The blue diamond indicates the *m*/*z* value of the precursor (417.9 Da).

Although NTA provides the specificity required for highly reliable peptide identification, it should always be followed by targeted validation [34]. Therefore, TA was performed using the multiple-reaction monitoring (MRM) method to quantify BPs by RP-ultrahigh-performance LC (RP-UHPLC) coupled to triple quadrupole MS (QQQ-MS) with an embedded electrospray ionisation (ESI) source. Peptide identification and spectral analysis were performed using MassHunter Workstation software, version B.08.00. Representative chromatograms and the MS spectrum of peptide ^213^TKVIPYVRYL^222^ are shown in Figure 4.

The identified peptide sequences were then searched against those in the Milk Bioactive Peptide Database (MBPD), the Database of Food-Derived Bioactive Peptides (DFBP) and the BIOPEP-UWM™ database to establish whether their bioactive properties have already been described in the scientific literature. Moreover, a manual literature search was performed to diminish retrieval bias. Of the 10 peptides detected by NTA in the 24-h sample, 5 peptides from α-s2- and β-casein exhibit the bioactive properties described in the scientific literature, while of the 20 peptides detected in the 48-h sample, 5 peptides from β- and κ-casein exhibit bioactive traits (Table 1). Two peptides identified in the 48-h sample, ^117^ARHPHPHLSFM^127^ and ^45^KYIPIQYVL^53^, were not detected in the 24-h sample, while two BPs (^209^IQPKTKVIPYVRYL^222^ and ^213^TKVIPYVRYL^222^) identified in the 24-h sample were not detected in the 48-h sample. It should be noted that the difference in only one amino acid may result in the loss of the bioactive properties of a peptide; e.g., the bioactive traits of peptides ^209^QEPVLGPVRGPFPIIV^224^ and ^117^ARHPHPHLSFM^127^ have been described in the literature, while the bioactive properties of peptides ^210^EPVLGPVRGPFPIIV^224^ and ^117^ARHPHPHLSF^126^ have not yet been recorded. Proteolysis of the peptide ^45^KYIPIQYVL^53^, which was detected after both 24 and 48 h of fermentation, led to the formation of its smaller κ-casein counterpart ^45^KYIPIQY^51^, which was detected after 48 h of fermentation with NTA and, in contrast to the peptide ^45^KYIPIQYVL^53^, has no bioactive properties described in the literature. The degradation of α-s2-casein by the proteolytic activity of the implemented strains led to the formation of the peptide ^209^IQPKTKVIPYVRYL^222^ and its smaller counterpart, the peptide ^213^TKVIPYVRYL^222^, both of which have bioactive properties. The longest peptide identified by NTA was the 24 amino acid-long ^63^YYQQKPVALINNQFLPYPYYAKPA^86^, which was only detected in the 24-h sample. After 48 h of fermentation, only its smaller counterparts, ^63^YYQQKPVALINN^74^, ^66^QKPVALINNQFLPYPYYAKPA^86^, ^74^NQFLPYPYYAKPA^86^ and ^75^QFLPYPYYAKPA^86^, were detected in the lyophilised samples. The presence of all BPs identified by NTA was confirmed by TA, and three additional BPs (^181^SQSKVLPVPQKAVPYPQ^197^, ^198^RDMPIQAF^205^ and ^207^LYQEPVLGPVRGPFPIIV^224^) with a broad spectrum of bioactive properties were also detected. The detection of additional BPs via TA was expected, as MRM is an extremely sensitive technique for the quantification of target peptides within complex mixtures, allowing for the simultaneous quantification of numerous proteins in parallel [35]. The molecular weights of the peptides released in this work ranged from 924.57 to 2889.49 Da (Table 1).

**Table 1:** Peptides identified by non-targeted (●) and targeted (●) analysis of peptides in lyophilised milk after 24 h and 48 h of fermentation by strains *Lc. lactis* subsp. *lactis* ZGBP5-51, *E. faecium* ZGBP5-52 and *E. faecalis* ZGBP5-53.

Precursor Protein	Peptide Sequence	FT	*m*/*z* [Da]	Biological Functions							Ref. No.
				ACE	AO	AM	AT	IM	OP	AC	
**α-s1-CN**	^40^VAPFPEVFGK^49^●	48 h	1090.61								
**κ-CN**	^117^ARHPHPHLSFM^127^●● *	48 h	1329.67	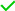	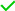						[36,37]
	^45^KYIPIQYVL^53^●●	both	1136.70		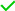						[38]
	^45^KYIPIQY^51^●	48 h	924.57								
	^54^SRYPSYGLN^62^●	48 h	1056.53								
	^54^SRYPSYGLNYY^64^●	48 h	1382.65								
	^63^YYQQKPVALINN^74^●	both	1450.80								
	^66^QKPVALINNQFLPYPYYAKPA^86^●	48 h	2435.28								
	^74^NQFLPYPYYAKPA^86^●	48 h	1571.83								
	^75^QFLPYPYYAKPA^86^●	both	1457.78								
	^87^AVRSPAQILQWQVL^100^●	both	1608.93								
	^117^ARHPHPHLSF^126^●	48 h	1198.64								
	^63^YYQQKPVALINNQFLPYPYYAKPA^86^●	24 h	2889.49								
**α-s2-CN**	^209^IQPKTKVIPYVRYL^222^●●	24 h	1718.04	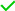	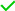						[39,40]
	^213^TKVIPYVRYL^222^●● *	24 h	1251.75			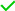					[41]
**β-CN**	^74^VYPFPGPIPN^83^●● *	both	1100.59	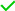	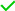				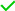		[40,42,43]
	^208^YQEPVLGPVRGPFPIIV^224^●● *	both	1881.07	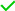	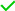	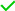	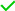	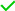		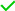	[42,44,45,46,47,48]
	^209^QEPVLGPVRGPFPIIV^224^●● *	both	1717.99	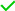	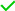						[42,49]
	^181^SQSKVLPVPQKAVPYPQ^197^● *	both	1865.04		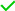					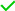	[40,50]
	^198^RDMPIQAF^205^● *	both	977.51	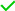							[44]
	^207^LYQEPVLGPVRGPFPIIV^224^● *	both	1994.18	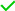							[51]
	^59^ELQDKIHPF^67^●	48 h	1126.60								
	^62^DKIHPFAQTQ^71^●	48 h	1184.62								
	^147^NLHLPLPLLQ^156^●	48 h	1157.72								
	^185^VLPVPQKAVPYPQ^197^●	48 h	1435.83								
	^210^EPVLGPVRGPFPIIV^224^●	48 h	1589.97								

**Abbreviations**:** FT**, fermentation time; **Ref. No.**, reference number; **α-s1-CN**, α-s1-casein; **α-s2-CN**, α-s2-casein; **β-CN**, β-casein; **κ-CN**, κ-casein; **ACE**, angiotensin I-converting enzyme inhibitory; **AO**, antioxidant; **AM**, antimicrobial; **AT**, antithrombotic; **IM**, immunomodulatory; **OP**, opioid; **AC**, anticancer. Asterisks (*) indicate peptides that were identified in the control samples of uninoculated, unfermented, pasteurised milk via a targeted peptide analysis. Superscript numbers before and after the peptide sequence indicate the position of the amino acid fragment in the precursor protein. The green tick (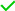) indicates that the biological function listed in the column has been demonstrated for the peptide listed in the row.

Of all BPs identified, seven (^209^IQPKTKVIPYVRYL^222^, ^74^VYPFPGPIPN^83^, ^208^YQEPVLGPVRGPFPIIV^224^, ^209^QEPVLGPVRGPFPIIV^224^, ^117^ARHPHPHLSFM^127^, ^198^RDMPIQAF^205^ and ^207^LYQEPVLGPVRGPFPIIV^224^) exhibit ACE inhibitory activity. ACE inhibitors are currently considered the first choice for the treatment of hypertension, which requires lifelong drug treatment. However, long-term administration of synthetic ACE inhibitors can be costly and is associated with various adverse side effects [52]. Therefore, there is growing interest in identifying ACE inhibitors derived from dietary proteins as an effective way to treat hypertension without incurring unacceptable side effects. The BIOPEP-UWM^TM^ database contains 1107 BPs with proven ACE inhibitory activity. Ibrahim et al. [36] highlighted that ^117^ARHPHPHLSFM^127^, the BP uncovered in this work, exerts a significant ACE inhibitory effect comparable to that of captopril, an antihypertensive drug, exhibiting an IC_50_ value of 4.27 µM, implying its potential therapeutic application. Six of the seven BPs discovered in this work with proven ACE inhibitory activity have proline, aromatic or aliphatic amino acid residues at the C-terminal position and aliphatic, basic or aromatic amino acid residues at the penultimate position, which is a feature of good ACE inhibitors [53,54]. The exception is the peptide ^74^VYPFPGPIPN^83^, which has a polar amino acid asparagine at the C-terminal position and proline at the penultimate position. Eisele et al. [42] reported that this peptide exhibits ACE inhibition and antioxidant activity with IC_50_ values of 325 and 6.2 μM, respectively, and therefore identified it as a weak ACE inhibitor and potent antioxidant peptide. The peptide ^198^RDMPIQAF^205^ also showed weak ACE inhibitory activity of IC_50_ = 197.46 [55]. Interestingly, Caira et al. [56] detected the presence of the ACE inhibitory peptides ^208^YQEPVLGPVRGPFPIIV^224^ and ^209^QEPVLGPVRGPFPIIV^224^ in human plasma after healthy individuals were given a cheese-enriched diet (100 g/day), highlighting the potential role of BP-fortified functional foods as nutraceuticals.

There are currently 837 peptide sequences with antioxidant activity registered in the BIOPEP-UWM^TM^ database, second only to ACE inhibitory peptides, underlining their great commercial value. Reactive oxygen species (ROS) are efficiently scavenged by antioxidant defence systems under normal conditions in vivo [57]. However, when the accumulation of ROS exceeds the capacity of the cellular free radical scavenging system, it triggers the impairment of non-target biomolecules and cells, mediates the subsequent activation of pro-inflammatory or pro-apoptotic metabolic pathways and triggers the onset and progression of many chronic diseases [58]. Accordingly, additional supplementation is required to regulate redox homeostasis and counteract the oxidative stress caused by free radicals. However, the long-term and high-dose use of synthetic antioxidants is strictly limited, as they exhibit varying degrees of cytotoxicity, carcinogenicity and endocrine disrupting effects [59]. In addition to their role as therapeutics, the research value of antioxidant BPs as food additives, as nutritional fortificators in functional foods and as anti-ageing and photoprotective components in cosmetics has also been studied in recent decades, as they directly scavenge free radicals, inhibit lipid peroxidation and chelate pro-oxidative transition metals, thus maintaining the stability of food flavour and nutritional quality [60,61]. Seven BPs detected in this work (^209^IQPKTKVIPYVRYL^222^, ^74^VYPFPGPIPN^83^, ^208^YQEPVLGPVRGPFPIIV^224^, ^209^QEPVLGPVRGPFPIIV^224^, ^117^ARHPHPHLSFM^127^, ^45^KYIPIQYVL^53^ and ^181^SQSKVLPVPQKAVPYPQ^197^) with proven antioxidant activity have between 9 and 17 mostly hydrophobic amino acid residues, which is consistent with Sonklin et al. [62], claiming that most antioxidant peptides have 2-to-20 mainly hydrophobic amino acid residues. Among them, the peptides ^74^VYPFPGPIPN^83^ and ^208^YQEPVLGPVRGPFPIIV^224^ exhibited radical scavenging activities, with IC_50_ values of 6.2 and 5.2 μM, respectively [42]. Tonolo et al. [37,40] proved that the peptides ^117^ARHPHPHLSFM^127^, ^74^VYPFPGPIPN^83^, ^181^SQSKVLPVPQKAVPYPQ^197^ and ^209^IQPKTKVIPYVRYL^222^ improve the survival of Caco-2 cells under oxidative stress.

As the treatment of bacterial infections has become a major clinical challenge due to the rapid development of antibiotic resistance, antimicrobial peptides (AMPs) from synthetic or natural sources have emerged as excellent candidates for overcoming antibiotic resistance, as they exhibit broad-spectrum antimicrobial activity coupled with high specificity, low toxicity and low propensity to develop resistance. The peptide ^213^TKVIPYVRYL^222^ showed antimicrobial activity against *Cronobacter sakazakii* and *Listeria monocytogenes* according to Alvarez-Ordóñez et al. [41], while the peptide ^208^YQEPVLGPVRGPFPIIV^224^ showed antibacterial activity against *Escherichia coli* DH5α [45].

Given the limitations of the available antithrombotic agents and in the search for a new generation of safe and effective antithrombotic alternatives with a broader therapeutic window, food-derived antithrombotic peptides have attracted attention as potential ingredients in health-promoting functional foods targeting thrombi due to their high biological activities, low toxicity and ease of metabolism in the human body [63]. The peptide ^208^YQEPVLGPVRGPFPIIV^224^, with proven antithrombotic bioactivity, was identified in this study. Rojas-Ronquillo et al. [46] reported the inhibition of fibrin cross-linking, which is responsible for the clotting activity, by the said peptide released from bovine casein during fermentation by *Lactobacillus casei* Shirota.

The peptide ^208^YQEPVLGPVRGPFPIIV^224^ also exerts an anti-infective immunostimulatory effect by upregulating the expression of major histocompatibility complex (MHC) class II antigens on bone marrow-derived macrophages from germ-free and human flora-associated mice, thereby increasing their phagocytic activity and inducing a low release of cytokines [47]. The importance of immunomodulatory peptides stems not only from their applicability in the food industry, medicine, cosmetics, pharmacology, etc., but also from their potential to attenuate various autoimmune diseases and their use in vaccines [64]. For example, the peptide QQPQDAVQPF, isolated from wheat, serves as an antagonist for the peptide from α-gliadin and could therefore be used to treat coeliac disease [65].

The peptide ^74^VYPFPGPIPN^83^, released from bovine β-casein in the present study, also exhibits opioid activity, in addition to ACE inhibitory and antioxidant activity [43]. Food-derived exogenous opioid peptides, also known as exorphins, could provide a safe alternative in the treatment of various stress-related diseases. They are mainly generated in the gastrointestinal tract, where they resist degradation by intestinal enzymes such as proteases, enter the bloodstream and cross the blood–brain barrier (BBB) to interact with opiate receptors [66].

Recently, anticancer BPs (ACPs) have emerged as potential therapeutics due to their high penetration, specificity and fewer side effects. The peptide ^208^YQEPVLGPVRGPFPIIV^224^ showed in vitro antiproliferative properties against the HT-29 colon cancer cell line by inducing apoptosis and cell cycle arrest in G2/M [48], while the peptide ^181^SQSKVLPVPQKAVPYPQ^197^ showed antiproliferative and anti-colon cancer effects on HT-29 cells [50]. Both ACPs identified in this work also exhibit antioxidant activity and could therefore apparently be used to alleviate the symptoms of oxidation-related diseases, including cancer.

To summarise, the detailed analysis and literature search revealed that the multifunctional BP ^208^YQEPVLGPVRGPFPIIV^224^ encompasses the broadest spectrum of bioactive properties of all BPs released from bovine milk fermented by strains ZGBP5-51, ZGBP5-52 and ZGBP5-53, ranging from ACE inhibitory, antioxidant, antimicrobial, antithrombotic and immunomodulatory to anticancer properties.

**Figure 5:**
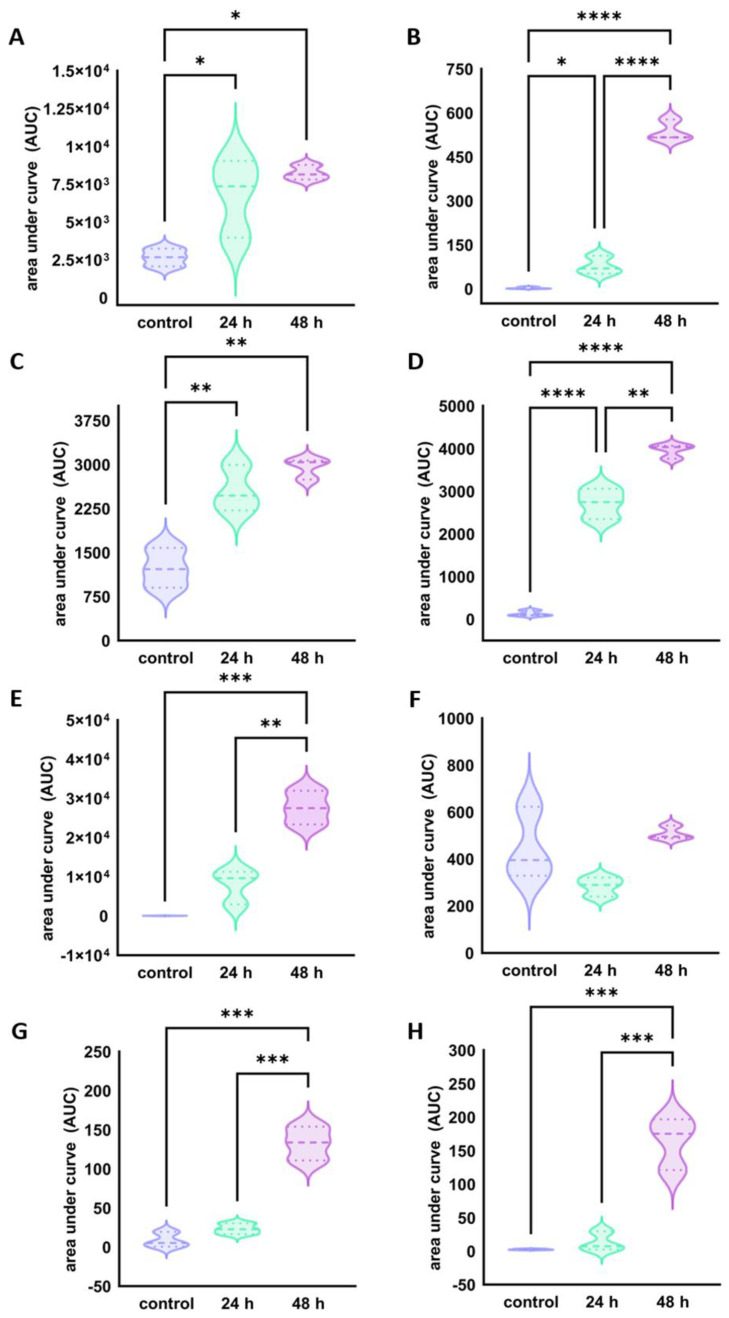
Graphical representation of the results of BP quantification by targeted MS analysis. The values of the area under the curve (AUC) correlate with the amount of each peptide ((**A**) ^213^TKVIPYVRYL^222^, (**B**) ^74^VYPFPGPIPN^83^, (**C**) ^208^YQEPVLGPVRGPFPIIV^224^, (**D**) ^209^QEPVLGPVRGPFPIIV^224^, (**E**) ^117^ARHPHPHLSFM^127^, (**F**) ^207^LYQEPVLGPVRGPFPIIV^224^, (**G**) ^181^SQSKVLPVPQKAVPYPQ^197^ and (**H**) ^198^RDMPIQAF^205^) quantified using the MRM method. Asterisks indicate AUC values for each peptide with significant difference (* *p* < 0.05, ** *p* < 0.01, *** *p* < 0.001 and **** *p* < 0.0001). The dashed lines (---) represent medians, while the dotted lines (⋯) represent the data quartiles.

The detected BPs were then quantified using the MRM method. A statistically significant (*p* < 0.05) increase in the amount of BPs detected after 48 h of milk fermentation by the selected strains compared to the control samples was observed for all peptides, with the exception of peptide ^207^LYQEPVLGPVRGPFPIIV^224^, of which a substantial amount was present in the control sample (448.90 ± 154.26), which decreased to 283.52 ± 40.95 after 24 h of fermentation and then increased again to 509.11 ± 29.66, but without significant meaning (Figure 5). Since the aforementioned peptide has the longest sequence of all quantified BPs, it is likely that the milk proteins degraded during storage of the milk, subsequently pilling the amount of peptide ^207^LYQEPVLGPVRGPFPIIV^224^ in the control sample (Figure 5F). After 24 h of fermentation by the implemented strains, there was a decrease (*p* = 0.16) in the amount of said peptide in the sample, as the proteolytic activity of the implemented strains degraded a certain amount of this peptide present in the control sample, resulting in a significant increase in the amount of other, smaller bioactive β-casein counterparts, i.e., ^208^YQEPVLGPVRGPFPIIV^224^ (*p* = 0.53 × 10^−2^) and ^209^QEPVLGPVRGPFPIIV^224^ (*p* = 0.22 × 10^−4^). The further truncation of peptide ^209^QEPVLGPVRGPFPIIV^224^ resulted in the appearance of its smaller β-casein counterpart, ^210^EPVLGPVRGPFPIIV^224^, which was identified by NTA in the 48-h sample and which had not previously been detected in the 24-h sample. In this case, the loss of only one amino acid resulted in a loss of bioactivity, and therefore, the peptide ^210^EPVLGPVRGPFPIIV^224^ was not quantified by TA. After 48 h of fermentation, the amount of peptide ^207^LYQEPVLGPVRGPFPIIV^224^ increased slightly (*p* = 0.06) as the strains continued to degrade proteins and peptides.

### 2.4. In Silico Analysis of Peptides Released from Bovine Milk Fermented by Strains ZGBP5-51, ZGBP5-52 and ZGBP5-53

**Figure 6:**
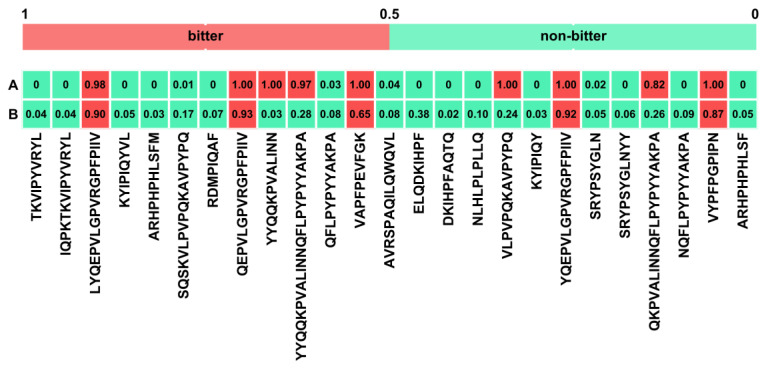
Peptide bitterness determined by (**A**) BERT4Bitter and (**B**) iBitter-Fuse computational models. Both tools were trained with a threshold of 0.5: any peptide with a predicted bitterness above a threshold of 0.5 was categorised as bitter.

Although food-derived BPs have significant bioactivity, their production at the industrial scale has been hampered by several challenges, such as the sustainability of resources for mass production, the low bioavailability and yield of the peptides, and the poor sensory performance of the peptides due to their bitterness [67]. The bitter-tasting hydrophobic peptides affect the sensory quality of the resulting products and limit their use in the food and pharmaceutical industries [68]. Since the identification of bitter peptides via experimental approaches is an expensive and time-consuming endeavour, the employment of in silico models in their identification is highly desirable [69]. Therefore, the bitterness of peptides was first assessed using the Bidirectional Encoder Representation from Transformers (BERT)-based model embedded on the BERT4Bitter website, which predicts bitter peptides solely based on their amino acid sequence. Using the above model, nine peptides (^207^LYQEPVLGPVRGPFPIIV^224^, ^209^QEPVLGPVRGPFPIIV^224^, ^63^YYQQKPVALINN^74^, ^63^YYQQKPVALINNQFLPYPYYAKPA^86^, ^40^VAPFPEVFGK^49^, ^158^VLPVPQKAVPYPQ^197^, ^208^YQEPVLGPVRGPFPIIV^224^, ^66^QKPVALINNQFLPYPYYAKPA^86^ and ^74^VYPFPGPIPN^83^) were identified as bitter (Figure 6A). Although Bert4Bitter provides a fairly high prediction accuracy, it has certain shortcomings: the authors assume that the embodiment of unimportant features in the model development could lead to information redundancy and overfitting [70]. Therefore, to enhance the performance of bitterness prediction, the improved iBitter-Fuse prediction model with the embedded Support Vector Machine (SVM)-based classifier was used, which yielded only five bitter peptides (^207^LYQEPVLGPVRGPFPIIV^224^, ^209^QEPVLGPVRGPFPIIV^224^, ^40^VAPFPEVFGK^49^, ^208^YQEPVLGPVRGPFPIIV^224^ and ^74^VYPFPGPIPN^83^), as shown in Figure 6B. The bitterness threshold of the peptide is influenced by the amino acid composition and sequence, the molecular weight and the critical spatial structure of the peptides. Approaches to reduce, mask and remove the bitter taste of protein hydrolysates include biological debittering (i.e., enzymatic hydrolysis, enzymatic deamidation and plastein reaction) and physicochemical debittering (i.e., selective separation, Maillard reaction and encapsulation) [68]. Spray drying is one of the most commonly used methods for masking unpleasant flavours, but it has certain disadvantages, as the high temperature can cause denaturation of the peptides and lead to a Maillard reaction [71]. In contrast, lyophilisation is carried out at low temperatures and under vacuum conditions, which is more suitable for heat-sensitive compounds. In addition to masking their bitter taste, lyophilisation improves the stability of BPs in protein hydrolysates and minimises the impact of processing on their physiological properties [72]. Therefore, lyophilisation played a dual role in this work: firstly, to mask the bitter flavour of the peptides released from the bovine milk fermented by the implemented strains; and secondly, to ensure the stability of the BPs in the prepared functional fermented dairy product, as well as the stability and shelf-life of the product itself.

Since the activity of BPs depends on both their physicochemical and structural properties [73], the amino acid composition; chemical formula; and physicochemical properties, such as the extinction coefficient, molecular weight, isoelectric point, net charge at neutral pH and grand average of hydropathy (GRAVY) value, were estimated using the NovoPro Peptide Property Calculator (Table 2). The theoretical molecular weights (924.09–2890.28 Da) of the peptides identified in this work are in approximate agreement with the experimental values listed in Table 1. A high variability in net charge (from −0.90 to 3.00) and pI values (5.22–11.05) was observed, indicating that, at the physiological pH (7.4), most peptides will be cationic or neutral, and only one peptide, ^59^ELQDKIHPF^67^, will be negatively charged. The characteristics of the peptides identified in this work were further analysed by their GRAVY index score. For globular proteins, a strong correspondence was observed between internal regions and negative (hydrophobic) GRAVY values and between external regions and positive (hydrophilic) GRAVY values, whereas for membrane-bound proteins, the parts of their sequences located within the lipid bilayer were correlated with hydrophobic areas [74]. The peptides ^117^ARHPHPHLSF^126^, ^54^SRYPSYGLN^62^, ^74^NQFLPYPYYAKPA^86^, ^54^SRYPSYGLNYY^64^, ^62^DKIHPFAQTQ^71^, ^59^ELQDKIHPF^67^, ^63^YYQQKPVALINNQFLPYPYYAKPA^86^, ^63^YYQQKPVALINN^74^, ^117^ARHPHPHLSFM^127^ and ^181^SQSKVLPVPQKAVPYPQ^197^ demonstrated strongly negative GRAVY values, which, according to Cid et al. [75], indicate a high hydrophilic character. The peptides ^207^LYQEPVLGPVRGPFPIIV^224^, ^45^KYIPIQYVL^53^, ^209^QEPVLGPVRGPFPIIV^224^ and ^147^NLHLPLPLLQ^156^ demonstrated strongly positive GRAVY scores, indicating a high hydrophobic character, while the rest of the peptides are characterised as amphipathic based on their GRAVY score. It is also evident from the results that all five peptides identified as bitter by the iBitter-Fuse tool (Figure 6B) have a neutral or positive net charge and are characterised as hydrophobic or amphipathic according to their GRAVY values. They also contain a high proportion of amino acids with higher hydrophobicity, such as tryptophan, isoleucine, tyrosine, phenylalanine, leucine, valine and proline, in particular, as well as several proline residues, at least one of which is located at the centre of the molecule. According to Liu et al. [68], a high proportion of hydrophobic amino acids, especially proline, promotes the formation of a strong bitter taste, especially when proline is located near the centre of the peptide molecule.

**Figure 7:**
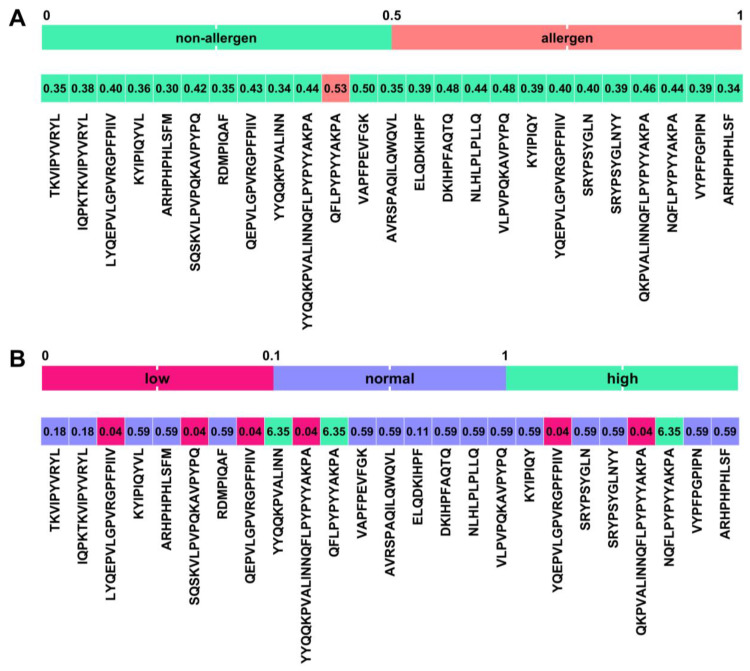
Prediction of (**A**) allergenicity and (**B**) half-life (s) of peptides identified in this work.

Using the AlgPred 2.0 web server, all peptides except ^75^QFLPYPYYAKPA^86^ were predicted to be non-allergens based on their amino acid sequence (Figure 7A). The half-life of the peptides was assessed using the HLP web server. According to the said server, most of the peptides identified in this work have a normal half-life (between 0.1 and 1.0 s). Longer peptides, i.e., ^181^SQSKVLPVPQKAVPYPQ^197^, ^63^YYQQKPVALINNQFLPYPYYAKPA^86^, ^207^LYQEPVLGPVRGPFPIIV^224^, ^208^YQEPVLGPVRGPFPIIV^224^, ^209^QEPVLGPVRGPFPIIV^224^ and ^66^QKPVALINNQFLPYPYYAKPA^86^, show a low half-life of less than 0.1, while the peptides ^63^YYQQKPVALINN^74^, ^75^QFLPYPYYAKPA^86^ and ^74^NQFLPYPYYAKPA^86^ demonstrated a high half-life.

During the development process of a new therapeutic drug, it is of utmost importance to know the fate of pharmacokinetics, i.e., the fate of a compound in the organism, as poor pharmacokinetic properties are one of the main causes of failures during clinical phases, in addition to a lack of efficacy and unacceptable toxicity [76]. This is usually established via individual indices called absorption, distribution, metabolism, excretion and toxicity (ADMET) parameters, which are usually determined using in silico modelling as an alternative to experimental methods. In this study, various ADMET parameters and physiochemical properties were estimated for all 25 peptides uncovered in this work, using ADMETlab 2.0 software, which is a comprehensive online database resource for drug metabolism and pharmacological data with 288,967 entries (Table 3). The central nervous system (CNS) permeability was determined using the BBB permeability score, which represents the ability of a molecule to penetrate the semipermeable BBB that protects the CNS from potentially harmful substances and excretes metabolic waste and, on the other hand, allows nutrients and some macromolecules required by brain tissue to pass through under physiological conditions. However, the existence of the BBB restricts the action of various bioactive compounds. The BBB permeability is crucial for the treatment of infectious conditions of the CNS, such as bacterial meningitis [77], and is therefore an extremely important feature of AMPs. All peptides uncovered in this work achieved an optimal BBB permeability score, including AMPs ^213^TKVIPYVRYL^222^ (0.02) and ^208^YQEPVLGPVRGPFPIIV^224^ (0.004). According to the free-drug principle, only the free (unbound) drug is able to diffuse across the membrane to achieve the therapeutic effect [78]. Drugs with high plasma protein binding (PPB), especially those with a narrow therapeutic window, are associated with serious side effects and safety concerns. Namely, a minor variation in drug–protein binding, stemming from the binding displacement of these drugs, can vastly increase the proportion of free drug and affect the therapeutic effects and side effects [79]. All peptides identified in this work have a favourable PPB, including the representative peptide ^213^TKVIPYVRYL^222^ (36.98%). The said peptide also has a favourable volume distribution (VD), i.e., the theoretical volume required for a drug to be evenly distributed in the blood (Table 3B). The higher the VD, the more a drug is distributed in the tissue rather than in the plasma. Also, all peptides identified in this work passed the in silico toxicity test, which consists of seven toxicophore rules. Toxicophores or structural alerts are molecular functionalities commonly associated with toxicity [80].

### 2.5. In Silico Identification of Potential Novel BPs Released during Microbial Fermentation of Bovine Milk by Strains ZGBP5-51, ZGBP5-52 and ZGBP5-53

**Figure 8:**
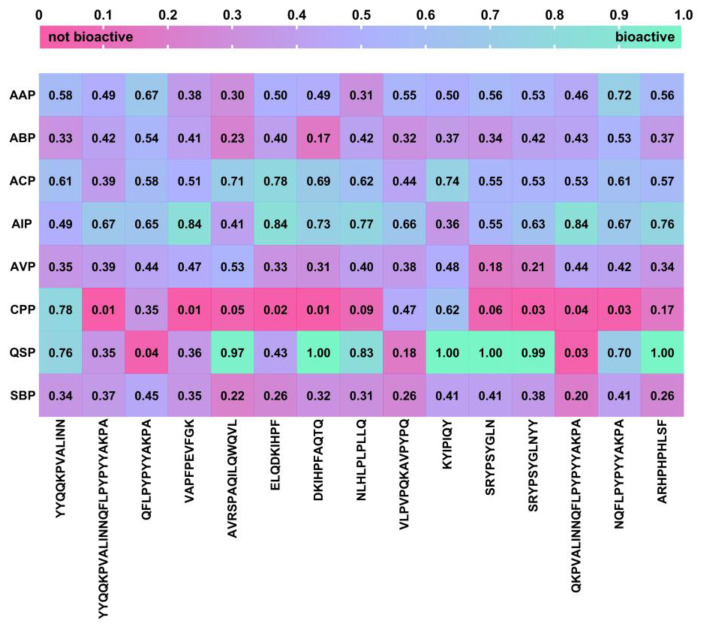
Heatmap of each bioactivity probability score (*ps*) obtained with the CSM-peptides platform. A *ps* of less than 0.7 indicates a negative therapeutic relevance, while a *ps* of more than 0.7 indicates that the peptide could belong to one of the eight different classes of therapeutic peptides: **AAP**, antiangiogenic peptide; **ABP**, antibacterial peptide; **ACP**, anticancer peptide; **AIP**, anti-inflammatory peptide; **AVP**, antiviral peptide; **CPP**, cell-penetrating peptide; **QSP**, quorum-sensing peptide; and **SBP**, surface-binding peptide.

As conventional methods of peptide characterisation are time-consuming and costly, the establishment of the bioinformatic analysis has opened up a new possibility to predict bioactivity or interaction with specific molecules and receptors via a homology-based search in specific databases and via molecular docking and structural alignments [81]. Therefore, a screening of the therapeutic potential of 15 peptides identified in this work, whose bioactive traits have not yet been described in the literature, was performed by combining the output of LC-MS-based peptidome profiling with two amino acid composition-based in silico bioactivity prediction tools to identify potential new BP candidates. First, the peptide sequences were subjected to the cutoff scanning matrix (CSM) platform, which, according to Rodrigues et al. [82], outperformed existing approaches for predicting the bioactivity of peptides such as PEPred, AI4ACP, FIRM-AVP and PPTPP by achieving an area under the curve (AUC) of up to 0.92 in independent blind tests and a consistent performance in cross-validation. The CSM-peptides platform is a powerful tool for the identification of therapeutic peptides from eight different classes: antiangiogenic (AAP), antibacterial (ABP), anticancer (ACP), cell penetrating (CPP), anti-inflammatory (AIP), antiviral (AVP), quorum sensing (QSP) and surface binding (SBP). The probability scores (*ps*) that peptides belong to one of these classes were visualised in a heatmap (Figure 8). Although this platform is trained with a default threshold of 0.5, the threshold was set to 0.7 to remove the false positives. With the adjusted threshold, 12 potential BPs were identified, some of which may have more than one therapeutic function. Among them, eight peptides were categorised as QSP, six as AIP, three as ACP, one as CPP and one as AAP. Peptide ^63^YYQQKPVALINN^74^ was categorised as a possible CPP, with *ps* = 0.78, and as a possible QSP (*ps* = 0.76); ^59^ELQDKIHPF^67^ as ACP (*ps* = 0.78) and AIP (*ps* = 0.84); peptide ^40^VAPFPEVFGK^49^ as AIP (*ps* = 0.84); ^87^AVRSPAQILQWQVL^100^ as ACP (*ps* = 0.71) and QSP (*ps* = 0.97); ^62^DKIHPFAQTQ^71^ as AIP (*ps* = 0.73) and QSP (*ps* = 1.00); ^147^NLHLPLPLLQ^156^ as AIP (*ps* = 0.77) and QSP (*ps* = 0.83); ^66^QKPVALINNQFLPYPYYAKPA^86^ as AIP (*ps* = 0.84); ^45^KYIPIQY^51^ as ACP (*ps* = 0.74) and QSP (*ps* = 1.00); ^54^SRYPSYGLN^62^ as QSP (*ps* = 1.00); ^54^SRYPSYGLNYY^64^ as QSP (*ps* = 0.99); ^74^NQFLPYPYYAKPA^86^ as AAP (*ps* = 0.72) and QSP (*ps* = 0.70); and ^117^ARHPHPHLSF^126^ as AIP (*ps* = 0.76) and QSP (*ps* = 1.00).

**Figure 9:**
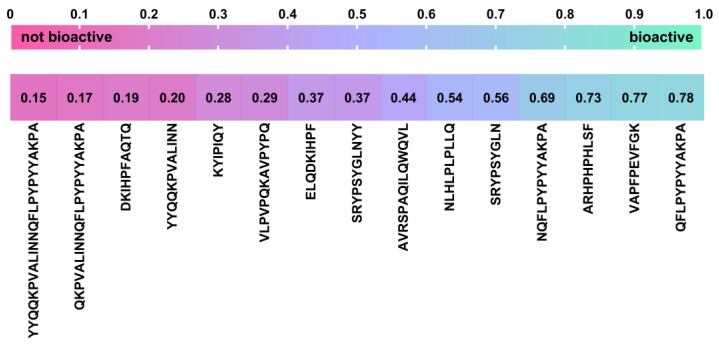
Heatmap of bioactivity probability scores (*ps*) determined by the PeptideRanker server based on a novel N-to-1 neural network. A *ps* of less than 0.7 indicates a negative therapeutic significance, while a *ps* of more than 0.7 indicates the therapeutic significance of a particular peptide.

The PeptideRanker server, which predicts BPs based on a novel N-to-1 neural network, was used to determine whether any of the peptides might have bioactive properties belonging to a class other than the eight classes covered by the CSM-peptides platform. The *ps* obtained were depicted as a heatmap in Figure 9. Although PeptideRanker was also trained with a threshold of 0.5, this was set to 0.7 to remove the false positives. According to Mooney et al. [83], increasing the threshold in PeptideRanker to 0.8 reduced the false-positive rate from 11% and 16% at a threshold of 0.5 to 2% and 6% at a threshold of 0.8 for long and short peptides, respectively. However, the authors declared that increasing the threshold to 0.8 also reduces the rate of true positives, so the threshold should be carefully chosen by researchers depending on whether it is more important to reduce the number of false positives or to capture all true positives. Also, a cysteine content of less than 4%, which is a feature of most peptides identified in this work, may lead to a false-negative result for a given peptide, which may be a reason why PeptideRanker predicted fewer potential BP candidates than the CSM-peptides platform. To be precise, with the adjusted threshold, only three peptides (^75^QFLPYPYYAKPA^86^, ^40^VAPFPEVFGK^49^ and ^117^ARHPHPHLSF^126^) were labelled as bioactive by the PeptideRanker tool, with probability scores of 0.78, 0.77 and 0.73, respectively, in contrast to 12 BPs predicted by the CSM-peptides platform with the same threshold. Among them, peptide ^40^VAPFPEVFGK^49^ was predicted by the CSM-peptides platform with the adjusted threshold as AIP (*ps* = 0.84) and with the default threshold as possible ACP (*ps* = 0.51), while peptide ^117^ARHPHPHLSF^126^ was classified as QSP (*ps* = 1.00) and AIP (*ps* = 0.76) with the adjusted threshold of 0.7 and as possible ACP (*ps* = 0.57) and AAP (*ps* = 0.56) with the default threshold of 0.5. Since peptides ^40^VAPFPEVFGK^49^ and ^117^ARHPHPHLSF^126^ were classified as possibly bioactive by both in silico bioactivity prediction tools, they should be further investigated as possible BP candidates, especially considering that peptide ^117^ARHPHPHLSF^126^ is only one amino acid residue shorter than its counterpart, ^117^ARHPHPHLSFM^127^, with already proven ACE inhibitory and antioxidant properties. Meanwhile, peptide ^75^QFLPYPYYAKPA^86^ was categorised as bioactive (*ps* = 0.78) by the PeptideRanker tool but did not fall into any therapeutic peptide category with the CSM-peptides platform when the threshold was set to 0.7, although it was assorted into four classes (AAP, ABP, ACP and AIP) of BPs by the same platform when the default threshold of 0.5 was considered. Moreover, there is a strong possibility that the said peptide has a bioactive property that falls out of the categories covered by the CSM-peptides platform. Therefore, the peptide ^75^QFLPYPYYAKPA^86^ should perhaps also be investigated as a possible BP candidate.

In summary, the genome analysis of strains ZGBP5-51, ZGBP5-52 and ZGBP5-53 revealed the diversity of their proteolytic systems, which contained a large number of genes (39, 45 and 46, respectively) that were involved in their proteolytic activity. The microbial fermentation of bovine milk by the joint application of the implemented strains proved to be a good method for the release of BPs, as it yielded a large number of peptides, 10 of which had proven bioactive properties. Moreover, by combining the output of MS-based peptidome profiling with in silico bioactivity prediction tools, three peptides (^75^QFLPYPYYAKPA^86^, ^40^VAPFPEVFGK^49^ and ^117^ARHPHPHLSF^126^), whose bioactive properties have not been previously reported in the literature, were revealed as potential BP candidates. Considering that enterococci represent an intriguing source of biologically active molecules of nutraceutical and therapeutic importance alongside BPs, but without forgetting their controversial status, the production of non-living enterococcal preparations known as postbiotics could represent a key to exploit their therapeutic effects in the future, without the risk of administering live microorganisms [84].

## 3. Materials and Methods

### 3.1. Bacterial Strains and Cultivation Conditions

In this study, the autochthonous strains *Lc. lactis* subsp. *lactis* ZGBP5-51, *E. faecium* ZGBP5-52 and *E. faecalis* ZGBP5-53 were used, which were isolated from artisanal Croatian fresh cheese. The strains were maintained as frozen stocks at −80 °C in M17 broth (Biolife Italiana S.r.l., Monza, Italy) supplemented with 15% (*v*/*v*) glycerol (Sigma-Aldrich, Saint Louis, MO, USA). Prior to use, strains were propagated in M17 broth and incubated overnight at 37 °C, under aerobic conditions.

### 3.2. WGS and Genome Annotation of Bacterial Strains

The genomic DNA of *Lc. lactis* subsp. *lactis* ZGBP5-51, *E. faecium* ZGBP5-52 and *E. faecalis* ZGBP5-53 was extracted using a Maxwell^®^ DNA Cell Kit in a Maxwell^®^ 16 Research System device (Promega, Madison, WI, USA), according to the manufacturer’s instructions. WGS was performed at IGA Technology Services (IGA Technology Services S.r.l., Udine, Italy), following the framework described in Banić et al. [85] but with slight modifications. The Celero^TM^ DNA-Seq Kit (NuGEN, San Carlos, CA, USA) was used for library preparation according to the manufacturer’s instructions. Both input and final libraries were quantified using a Qubit 2.0. Fluorometer (Invitrogen, Carlsbad, CA, USA) and quality tested using the Agilent 2100 Bioanalyzer High Sensitivity DNA Assay (Agilent Technologies, Santa Clara, CA, USA). The resulting libraries were then sequenced on Illumina NovaSeq 6000 (Illumina, San Diego, CA, USA) in paired-end 250 mode, generating 3 million reads for each sample. The bcl2fastq v2.20. version of the Illumina pipeline was used to process the raw data for both format conversion and demultiplexing to remove low-quality bases [86]. Adapter sequences were masked with Cutadapt v1.11, using default parameters, but with -O 5-n 2-m 35. Quality trimming was performed using ERNE v1.4.6 with default parameters. The trimmed reads were assembled using SPAdes v3.14.1 with the default parameters, but with -m 200-k 21-, 33-, 55- and 77-isolate de novo. The assembled contigs were polished with the Illumina reads by aligning them with the reference sequence with BWA mem v0.7.17, and calling homozygous SNPs and indels were determined with pilon v1.23. The consensus sequence was generated with bcftools v1.9. Contigs were classified when receiving the best blastn v2.14.0 hit with a minimum e-value of 1 × 10^−5^ in the NCBI nt database (https://blast.ncbi.nlm.nih.gov/Blast.cgi, accessed on 23 March 2023) [87]. This Whole Genome Shotgun Project was submitted to the NCBI nt database (BioProject PRJNA769625). Whole-genome sequences of the implemented strains *Lc. lactis* subsp. *lactis* ZGBP5-51, *E. faecium* ZGBP5-52 and *E. faecalis* ZGBP5-53 are available in GenBank under the accession numbers JAXBVH000000000 (BioProject PRJNA388578, Biosample SAMN38281847), JAXBVG000000000 (BioProject PRJNA388578, Biosample SAMN38282040) and JAXCGY000000000 (BioProject PRJNA388578, Biosample SAMN38380667), respectively. The assembled genomes were then annotated using the BV-BRC system (https://www.bv-brc.org/, accessed on 24 March 2023) and analysed in silico, using the same system for the presence of genes involved in the proteolytic activity of the implemented strains, as well as for the possible presence of AMR phenotypes and common virulence factors [88]. Circular genome maps were then generated using the Circular Viewer functionality integrated into the BV-BRC system.

### 3.3. Preparation of the Functional Fermented BP-Rich Lyophilised Dairy Product

The strains *Lc. lactis* subsp. *lactis* ZGBP5-51, *E. faecium* ZGBP5-52 and *E. faecalis* ZGBP5-53 were routinely propagated in M17 broth and incubated at 37 °C, under aerobic conditions, until the optical density (OD) at 620 nm reached 1.0. To prepare the LAB consortium used for the production of a functional fermented lyophilised dairy product, the prepared cultures were mixed in a 1:1:1 ratio (*v*/*v*/*v*), and the bacterial cells were harvested by centrifugation at 4200 rpm for 5 min. The cell pellet was then washed in sterile distilled water and suspended in 12 mL of 3.2% (*w*/*v*) fresh milk (Mini Dairy-Veronika, Desinić, Croatia). The cell suspension was added to the milk as 4% (*v*/*v*) inoculum, and 50 mL of thus prepared samples was incubated in three replicates at 37 °C for 24 and 48 h, respectively. The unfermented, uninoculated, pasteurised milk served as a control. The pH and titratable acidity (°SH) of all samples were determined before and after the 24- and 48-h fermentation, as previously described by Leboš Pavunc et al. [89]. Cell viability was determined by an indirect method, i.e., by counting the number of colony-forming units (CFUs) that developed on M17 agar (Biolife Italiana S.r.l., Monza, Italy), using a standard pour plate technique. To produce a lyophilised dairy product, the fermented milk and control samples were frozen overnight at −80 °C and freeze-dried in the Christ Alpha 1-2 LD plus freeze-dryer (Martin Christ Gefriertrocknungsanlagen GmbH, Osterode, Germany). The BPs released during the microbial fermentation of bovine milk were then identified, characterised and quantified by non-targeted (NTA) or targeted (TA) peptide analysis of the prepared lyophilised dairy products.

### 3.4. Peptidomic Profiling of BP-Rich Lyophilised Dairy Product

#### 3.4.1. Non-Targeted Peptide Analysis (NTA)

##### Preparation of the Samples

The lyophilised samples were dissolved in 95/5 (*v*/*v*) 0.1% (*v*/*v*) trifluoroacetic acid/acetonitrile (TFA/ACN; VWR International, Radnor, PA, USA) to a final concentration of 1 mg/mL. To dissolve the solid particles, the prepared solutions were briefly vortexed, sonicated for 30 min in an ultrasonic bath (FALC Instruments S.r.l., Treviglio, Italy) at room temperature and filtered for 5 min at 10,000 rpm through a Nanosep MF centrifugal device (Pall Corporation, Port Washington, NY, USA) with a 0.2 µm wwPTFE membrane (Pall Corporation, Port Washington, NY, USA) at room temperature. Finally, 100 µL of each prepared filtrate was added to a LC vial (Agilent Technologies, Inc., Santa Clara, CA, USA).

##### RP-Nano LC Workflow

Peptides were separated using the Dionex UltiMate 3000 RSLC Gradient RP nano-LC system (Thermo Fisher Scientific, Waltham, MA, USA) with a UV/VIS detector coupled to the Proteineer fc II spotter (Bruker Corporation, Billerica, MA, USA). Peptide purification was performed on an Acclaim PepMap 100 C18, 5 µm, 100 Å, 300 µm i.d. × 5 mm precolumn (Thermo Fisher Scientific, Waltham, MA, USA) at 40 °C and with a 10 µL/min flow rate. The isocratic elution was programmed to increase over 5 min. Chromatographic separation of the peptides was performed on an Acclaim PepMap 100 C18, 3 µm, 100 Å, 75 µm i.d. × 15 cm column (Thermo Fisher Scientific, Waltham, MA, USA) at 40 °C and with a 0.3 µL/min flow rate. In both columns, mobile phase A consisted of 0.1% (*v*/*v*) aqueous TFA solution in 2% (*v*/*v*) ACN and mobile phase B consisted of 0.1% (*v*/*v*) TFA in 2% (*v*/*v*) ACN. The elution gradient was programmed to increase from 2 to 90% (*v*/*v*) over a period of 70 min with solvent B and the column was then conditioned back to the initial conditions. The total run time was 75 min. The detection wavelength of the detector was set to 215 nm. The flow rate of the spotter was set to 100 µL/h. The fractions were then collected on a MALDI target plate.

##### MALDI-TOF/TOF MS

Tandem MS was performed using an Autoflex speed MALDI-TOF/TOF analyser (Bruker Corporation, Billerica, MA, USA). The parameters for MS and MS/MS acquisition were optimised using WARP-LC version 1.3 software (Bruker Corporation, Billerica, MA, USA). External calibration of the spectra was performed using a cubic enhanced algorithm provided by the instrument software. Mass spectra were obtained in positive ion-reflectron mode in a mass range of *m*/*z* 1000–3500 Da. An α-cyano-4-hydroxycinnamic acid (CHCA) matrix was used to facilitate peptide ionisation. Tandem MS (MS/MS) analysis was performed with the following parameters: signal-to-noise (S/N) threshold of 10, 100 ppm mass tolerance between compounds, merging compounds separated by less than 6 fractions, and 5.0 Da as minimum mass distance to co-eluting compounds.

##### Peptide Identification

The database search was performed with ProteinScape 3.0 (Bruker Daltonics, Bremen, Germany), which uses the Mascot 2.5.0 algorithm (Matrix Science, London, UK) to match peptide peaks with proteins in the SwissProt database (Swiss Institute of Bioinformatics, Lausanne, Switzerland). The following search parameters were set for the Mascot search: *Bos taurus* taxonomy, variable modifications not specified, non-specific cleavage, 100 ppm precursor mass tolerance and 0.5 Da fragment mass tolerance.

#### 3.4.2. Targeted Peptide Analysis (TA) Using MS-Based MRM Method

TA consisting of RP-UHPLC coupled with QQQ-MS with an embedded ESI source was used for the relative quantification of previously identified BPs in milk samples fermented with *Lc. lactis* subsp. *lactis* ZGBP5-51, *E. faecium* ZGBP5-52 and *E. faecalis* ZGBP5-53. The TA was performed according to Novak et al. [90], with slight modifications. Optimisation of the MRM method used for the quantification of BPs was performed using Skyline software version 21.1.0.278 [91]. Lyophilised samples were diluted in 5/0.1/95 ACN/formic acid (FA)/water to a final concentration of 1 mg/mL. The prepared solutions were briefly vortexed; sonicated for 30 min in an ultrasonic bath at room temperature; and filtered for 5 min at 10,000 rpm, through a Nanosep MF centrifugal device with a 0.2 µm wwPTFE membrane, at room temperature, to remove undissolved particles from the samples, which could clog the column even at low concentrations and lead to high back pressure in the device. Finally, 100 µL of each prepared filtrate was added to an LC vial. Samples were analysed using the 1290 Infinity UHPLC system (Agilent Technologies, Santa Clara, CA, USA) coupled to the Agilent 6460 Triple Quad mass spectrometer with ESI source. Ionisation was performed in positive working mode (ESI+). The ion source parameters were set as follows: capillary voltage of 3.5 kV, sheath gas temperature of 300 °C, sheath gas flow of 9 L/min, gas flow of 7 L/min, and nebuliser pressure of 40 psi. An Acquity UPLC BEH C18 (2.1 mm × 50 mm × 1.7 µm, 130 Å) column (Waters Corporation, Milford, MA, USA) was used for chromatographic separation. The flow rate and temperature of the column were set to 0.3 mL/min and 40 °C, respectively. Mobile phase A consisted of 0.1% (*v*/*v*) aqueous FA, while mobile phase B consisted of 0.1% (*v*/*v*) FA in ACN. Peak identification and spectral analysis were performed using MassHunter Workstation software version B.08.00 (Agilent Technologies, Santa Clara, CA, USA). All peptide sequences identified and quantified by NTA or TA were matched with peptide sequences stored in the MBPD (https://mbpdb.nws.oregonstate.edu/, accessed on 28 August 2023) [92], DFBP (http://www.cqudfbp.net, accessed on 29 August 2023) [93] and the BIOPEP-UWM™ (https://biochemia.uwm.edu.pl/biopep/start_biopep.php, accessed on 30 August 2023) databases [94] to determine whether their bioactive properties have already been described in the scientific literature. In addition, a manual literature search was performed to minimise bias in the search.

### 3.5. In Silico Analyses

#### 3.5.1. In Silico Analysis of Peptides Released from Bovine Milk Fermented by Strains ZGBP5-51, ZGBP5-52 and ZGBP5-53

First, the bitterness of the peptides was appraised using BERT4Bitter (http://pmlab.pythonanywhere.com/BERT4Bitter, accessed on 12 September 2023) [69] and iBitter-Fuse (https://camt.pythonanywhere.com/iBitter-Fuse, accessed on 13 September 2023) [70]. Both tools were trained with a threshold of 0.5: any peptide with a predicted bitterness above a threshold of 0.5 was categorised as bitter. Next, the amino acid composition; chemical formula; and physicochemical properties, such as the extinction coefficient, molecular weight, isoelectric point, net charge at neutral pH, isoelectric point and GRAVY, were evaluated using the NovoPro Peptide Property Calculator (https://www.novoprolabs.com/tools/calc_peptide_property, accessed on 14 September 2023). The half-life of the peptides was analysed with the HLP web server (https://webs.iiitd.edu.in/raghava/hlp/, accessed on 7 February 2024). If the half-life in seconds was less than 0.1, the stability of the peptides was classified as low. If the half-life was between 0.1 and 1.0 s, the peptides were defined as normal, and if it was higher than 1.0, the peptides were categorised as high. The allergenicity of the peptides was assessed using the AlgPred 2.0 web server (https://webs.iiitd.edu.in/raghava/algpred2/index.html, accessed on 7 February 2024) on the basis of their amino acid composition. The threshold was set at 0.5: any peptide with a predicted allergenicity above a threshold of 0.5 was categorised as an allergen. Finally, the PepSMI tool (https://www.novoprolabs.com/tools/convert-peptide-to-smiles-string, accessed on 7 August 2023) was used to convert peptide sequences into SMILES strings, which were then analysed by ADMETlab 2.0 (https://admet.scbdd.com/home/index/, accessed on 15 September 2023) [95].

#### 3.5.2. In Silico Identification of Potential Novel BPs Released during the Fermentation of Bovine Milk by the Strains *Lc. lactis* subsp. *lactis* ZGBP5-51, *E. faecium* ZGBP5-52 and *E. faecalis* ZGBP5-53

For rapid in silico identification of potential BP candidates whose bioactive properties have not yet been described in the literature, a screening of the therapeutic potential of all peptides identified by NTA and TA was performed using two bioactivity prediction tools. First, the CSM-peptides platform (https://biosig.lab.uq.edu.au/csm_peptides/, accessed on 11 September 2023) [82] was used to identify potential BPs based on their amino acid composition. Next, the PeptideRanker server (http://distilldeep.ucd.ie/PeptideRanker/, accessed on 12 September 2023) [83] was used to predict BPs based on a novel N-to-1 neural network (N1-NN). Although both tools were trained with a threshold of 0.5, the researchers decided to set the threshold to 0.7 to avoid false-positive results. Therefore, any peptide predicted above a threshold of 0.7 was categorised as bioactive.

### 3.6. Graphical Representation and Statistical Analysis

All graphs, calculations and statistical analyses were performed with GraphPad Prism (GraphPad Software, Inc., San Diego, CA, USA) version 10.1.1. To assess the significance of differences between multiple pairs of means in the data group, an ordinary one-way analysis of variance (ANOVA) test was used, and Tukey’s multiple comparisons “post-hoc” test was applied. Statistical differences between groups were considered significant if the *p*-values were less than 0.05.

## Figures and Tables

**Table 2:** Amino acid composition and physicochemical properties of peptides released from bovine milk fermented by *Lc. lactis* subsp. *lactis* ZGBP5-51, *E. faecium* ZGBP5-52 and *E. faecalis* ZGBP5-53.

AA Composition	Chemical Formula	Physiochemical Properties				
		GRAVY	Net Charge (pH = 7)	pI	MW [Da]	ε [L/mol cm]
H- T K VIP Y V R Y L -OH	C_61_H_98_N_14_O_14_	0.34	2.00	10.04	1251.51	2560
H- I Q P K T K VIP Y V R Y L -OH	C_83_H_136_N_20_O_19_	−0.08	3.00	10.34	1718.08	2560
H- L YQ E PVL G PV R G PFPIIV -OH	C_97_H_152_N_22_O_23_	0.67	0.00	7.00	1994.37	1280
H- K Y IPI QY VL -OH	C_57_H_89_N_11_O_13_	0.60	1.00	9.52	1136.38	2560
H- A RH P H P H L S FM -OH	C_60_H_88_N_20_O_13_S	−0.71	1.30	11.05	1329.53	0
H- SQS K VLPVP Q K AVP Y P Q -OH	C_86_H_140_N_22_O_24_	−0.55	2.00	10.18	1866.16	1280
H- R D MPI Q AF -OH	C_43_H_68_N_12_O_12_S	−0.26	0.00	7.00	977.13	0
H- Q E PVL G PV R G PFPIIV -OH	C_82_H_132_N_20_O_20_	0.59	0.00	7.00	1718.04	0
H- YYQQ K PVALI NN -OH	C_67_H_103_N_17_O_19_	−0.65	1.00	9.52	1450.63	2560
H- YYQQ K PVALI NNQ FLP Y P YY A K PA -OH	C_141_H_201_N_31_O_35_	−0.57	2.00	9.57	2890.28	6400
H- Q FLP Y P YY A K PA -OH	C_74_H_100_N_14_O_17_	−0.49	1.00	9.39	1457.66	3840
H- VAPFP E VF G K -OH	C_54_H_79_N_11_O_13_	0.48	0.00	7.00	1090.27	0
H- AV R S PA Q IL Q W Q VL -OH	C_74_H_121_N_21_O_19_	0.41	1.00	11.05	1608.87	5690
H- E L Q D K I H PF -OH	C_52_H_79_N_13_O_15_	−0.90	−0.90	5.22	1126.26	0
H- D K I H PFA QTQ -OH	C_53_H_81_N_15_O_16_	−1.08	0.10	7.88	1184.29	0
H- N L H LPLPLL Q -OH	C_55_H_92_N_14_O_13_	0.56	0.10	7.88	1157.40	0
H- VLPVP Q K AVP Y P Q -OH	C_69_H_110_N_16_O_17_	−0.03	1.00	9.72	1435.70	1280
H- K Y IPI QY -OH	C_46_H_69_N_9_O_11_	−0.37	1.00	9.52	924.09	2560
H- YQ E PVL G PV R G PFPIIV -OH	C_91_H_141_N_21_O_22_	0.48	0.00	7.00	1881.21	1280
H- S R Y P SYG L N -OH	C_47_H_69_N_13_O_15_	−1.16	1.00	9.58	1056.12	2560
H- S R Y P SYG L NYY -OH	C_65_H_87_N_15_O_19_	−1.18	1.00	9.32	1382.47	5120
H- Q K PVALI NNQ FLP Y P YY A K PA -OH	C_118_H_175_N_27_O_29_	−0.36	2.00	9.78	2435.80	3840
H- NQ FLP Y P YY A K PA -OH	C_78_H_106_N_16_O_19_	−0.72	1.00	9.39	1571.76	3840
H- V Y PFP G PIP N -OH	C_55_H_77_N_11_O_13_	−0.01	0.00	7.00	1100.26	1280
H- A RH P H P H L S F -OH	C_55_H_79_N_19_O_12_	−0.97	1.30	11.05	1198.33	0

**Abbreviations: AA**, amino acid; **GRAVY**, grand average of hydropathy; **pI**, isoelectric point (theoretical); **MW**, molecular weight (theoretical); **ε**, extinction coefficient; **X**, hydrophobic uncharged residues, like F, I, L, M, V, W, A and P; **X**, basic residues, like R-, K-, H- and N-terminal -NH2; **X**, acidic residues, like D-, E-, and C-terminal -COOH; **X**, polar uncharged residues, like G, S, T, C, N, Q and Y.

**Table 3:** (**A**) 2D structure, physiochemical properties and (**B**) ADMET properties of peptides identified after microbial fermentation of bovine milk.

**(A)** 2D Structure and Physiochemical Properties of the Representative ^213^TKVIPYVRYL^222^ Peptide																				
**2D structure**																				
** 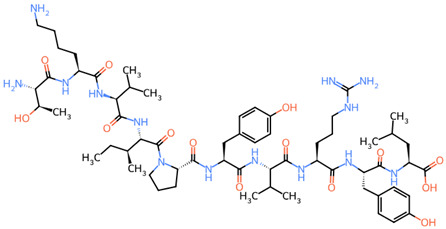 **																				
**Radar chart of physicochemical properties**																				
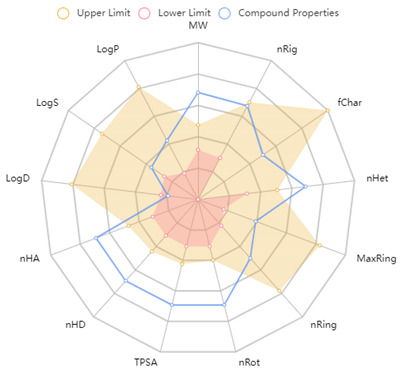												**MW**				1250.74				
												**nRig**				28				
												**fChar**				0				
												**nHet**				28				
												**MaxRing**				6				
												**nRing**				3				
												**nRot**				47				
												**TPSA**				465.04				
												**nHD**				20				
												**logD**				0.84				
												**logS**				−3.06				
												**logP**				1.14				
**(B)** ADMET properties of peptides identified after fermentation of bovine milk by strains ZGBP5-51, ZGBP5-52 and ZGBP5-53.																				
**Peptide**	**PPB**		**VD**		**BBB** **penetration**		**Acute toxicity**		**Genotoxic** **carcinogenicity**		**Nongenotoxic** **carcinogenicity**		**Skin sensitisation**		**Aquatic toxicity**		**Nonbiodegradable rule**		**SureCheMBL Rule**	
	**Value [%]**	**Decision**	**Value**	**Decision**	**Value**	**Decision**	**Alerts**	**Decision**	**Alerts**	**Decision**	**Alerts**	**Decision**	**Alerts**	**Decision**	**Alerts**	**Decision**	**Alerts**	**Decision**	**Alerts**	**Decision**
**1**	36.98	●	0.55	●	0.02	●	0	●	0	●	0	●	2	●	0	●	0	●	0	●
**2**	36.98	●	0.43	●	0.04	●	0	●	0	●	0	●	1	●	0	●	0	●	0	●
**3**	61.6	●	0.62	●	0.01	●	0	●	0	●	0	●	2	●	0	●	0	●	0	●
**4**	65.45	●	0.55	●	0.02	●	0	●	0	●	0	●	2	●	0	●	0	●	0	●
**5**	74.45	●	0.64	●	0.00	●	0	●	0	●	0	●	1	●	0	●	0	●	0	●
**6**	70.82	●	0.63	●	0.01	●	0	●	0	●	0	●	1	●	0	●	0	●	0	●
**7**	15.64	●	0.53	●	0.04	●	0	●	0	●	0	●	1	●	0	●	0	●	0	●
**8**	35.64	●	0.46	●	0.01	●	0	●	0	●	0	●	2	●	0	●	0	●	0	●
**9**	69.99	●	0.48	●	0.01	●	0	●	0	●	0	●	1	●	0	●	0	●	0	●
**10**	73.47	●	0.64	●	0.00	●	0	●	0	●	0	●	1	●	0	●	0	●	0	●
**11**	32.33	●	0.59	●	0.02	●	0	●	0	●	0	●	2	●	0	●	0	●	0	●
**12**	71.34	●	0.62	●	0.00	●	0	●	0	●	0	●	2	●	0	●	0	●	0	●
**13**	65.89	●	0.58	●	0.01	●	0	●	0	●	0	●	2	●	0	●	0	●	0	●
**14**	37.73	●	0.50	●	0.02	●	0	●	0	●	0	●	2	●	0	●	0	●	0	●
**15**	50.29	●	0.50	●	0.02	●	0	●	0	●	0	●	1	●	0	●	0	●	0	●
**16**	21.57	●	0.61	●	0.04	●	0	●	0	●	0	●	2	●	0	●	0	●	0	●
**17**	21.16	●	0.47	●	0.05	●	0	●	0	●	0	●	2	●	0	●	0	●	0	●
**18**	48.75	●	0.53	●	0.03	●	0	●	0	●	0	●	1	●	0	●	0	●	0	●
**19**	51.86	●	0.63	●	0.01	●	0	●	0	●	0	●	2	●	0	●	0	●	0	●
**20**	52.89	●	0.50	●	0.03	●	0	●	0	●	0	●	2	●	0	●	0	●	0	●
**21**	23.16	●	0.52	●	0.05	●	0	●	0	●	0	●	1	●	0	●	0	●	0	●
**22**	53.51	●	0.61	●	0.02	●	0	●	0	●	0	●	1	●	0	●	0	●	0	●
**23**	68.15	●	0.59	●	0.00	●	0	●	0	●	0	●	2	●	0	●	0	●	0	●
**24**	62.79	●	0.60	●	0.01	●	0	●	0	●	0	●	2	●	0	●	0	●	0	●
**25**	36.75	●	0.41	●	0.07	●	0	●	0	●	0	●	1	●	0	●	0	●	0	●

**Abbreviations**:** MW**, molecular weight; **nRig**, number of rigid bonds; **fChar**, formal charge; **nHet**, number of heteroatoms; **MaxRing**, number of atoms in the biggest ring; **nRing**, number of rings; **nRot**, number of rotatable bonds; **TPSA**, Topological Polar Surface Area; **nHD**, number of hydrogen bond donors; **logD**, logP at physiological pH 7.4; **logS**, log of the aqueous solubility; **logP**, log of the octanol/water portion coefficient. **PPB**, plasma protein binding; **VD**, volume distribution; **BBB**, blood–brain barrier; **1**, ^213^TKVIPYVRYL^222^; **2**, ARHPHPHLSFM; **3**, ^209^IQPKTKVIPYVRYL^222^; **4**, ^45^KYIPIQYVL^53^; **5**, ^207^LYQEPVLGPVRGPFPIIV^224^; **6**, ^209^QEPVLGPVRGPFPIIV^224^; **7**, ^198^RDMPIQAF^205^; **8**, ^181^SQSKVLPVPQKAVPYPQ^197^; **9**, ^74^VYPFPGPIPN^83^; **10**, ^208^YQEPVLGPVRGPFPIIV^224^; **11**, ^63^YYQQKPVALINN^74^; **12**, ^63^YYQQKPVALINNQFLPYPYYAKPA^86^; **13**, ^75^QFLPYPYYAKPA^86^; **14**, ^40^VAPFPEVFGK^49^; **15**, ^87^AVRSPAQILQWQVL^100^; **16**, ^59^ELQDKIHPF^67^; **17**, ^62^DKIHPFAQTQ^71^; **18**, ^147^NLHLPLPLLQ^156^; **19**, ^185^VLPVPQKAVPYPQ^197^; **20**, ^45^KYIPIQY^51^; **21**, ^54^SRYPSYGLN^62^; **22**, ^54^SRYPSYGLNYY^64^; **23**, ^66^QKPVALINNQFLPYPYYAKPA^86^; **24**, ^74^NQFLPYPYYAKPA^86^; **25**, ^117^ARHPHPHLSF^126^; ● favourable outcome; ● acceptable outcome.

## Data Availability

Data is contained within the article and supplementary material.

## Supplementary Materials

The supporting information can be downloaded at https://www.mdpi.com/article/10.3390/ijms25042431/s1.
