# Supplementary material for: An Integrated Comprehensive Peptidomics and In Silico Analysis of Bioactive Peptide-Rich Milk Fermented by Three Autochthonous Cocci Strains
Source: Int J Mol Sci. 2024 Feb 19;25(4):2431. doi: 10.3390/ijms25042431 (PMC10888711; doi:10.3390/ijms25042431)
Supplement: Supplementary file 1 [file ijms-25-02431-s001.zip › ijms-2843999-supplementary.pdf]

Table S1. Genes of the proteolytic system of strains **A** *Lactococcus lactis* subsp. *lactis* ZGBP5-51, **B** *Enterococcus faecium* ZGBP5-52 and **C** *Enterococcus faecalis* ZGBP5-53 used for the production of BP-rich lyophilised dairy products

A *Lactococcus lactis* subsp. *lactis* ZGBP5-51

Product type	Start	End	Strand	AA Lenght	Product
AP	111769	112836	-	355	Glutamyl aminopeptidase
	22827	25367	+	846	Lysyl aminopeptidase
	176368	177426	-	352	Aminopeptidase YpdF (MP-, MA-, MS-, AP-, NP- specific)
	71050	71904	-	284	Methionine aminopeptidase
	9166	10407	-	413	Tripeptide aminopeptidase
	11409	12773	+	454	Aminopeptidase C
	3	761	+	252	Aminopeptidase S (Leu, Val, Phe, Tyr preference)
	868	1347	-	159	Putative aminopeptidase YsdC
PR	8597	9889	+	430	Uncharacterized protease YrrO
	21658	22686	+	342	Lon-like protease with PDZ domain
	15142	16428	+	428	Intramembrane protease RasP/YluC, implicated in cell division
	9990	11216	-	408	Serine protease, DegP/HtrA, do-like
	6547	7314	+	255	Prophage Clp protease-like protein
	1688	1837	+	49	Epidermin leader peptide processing Ser protease epiP precursor
	4938	5645	+	235	Phage head, head-tail preconnector protease C
	2280	2870	+	196	Phage head maturation protease
	3262	3930	-	222	CAAX amino terminal protease family protein
	1599	2102	-	167	Intracellular protease
	1687	2370	-	227	Intracellular protease
	3	881	+	292	Protease IV
EP	3	320	-	106	Neutral endopeptidase
	3	206	+	67	Neutral endopeptidase
	3	320	-	106	Neutral endopeptidase
	1	1512	-	504	Neutral endopeptidase
	1266	1469	-	67	Neutral endopeptidase
P	109306	111711	+	801	Multimodular transpeptidase-transglycosylase
	171541	172920	+	459	Dipeptidase
	88489	88941	+	150	Lipoprotein signal peptidase
	103043	105274	-	743	Multimodular transpeptidase-transglycosylase
	64484	66775	+	763	Xaa-Pro dipeptidyl-peptidase
	90084	92078	-	664	Multimodular transpeptidase-transglycosylase/Penicillin-binding protein 1A/1B (PBP1)
	7114	8037	+	307	Peptidase, U32 family small subunit [C1]
	45802	47226	+	474	Dipeptidase
	4250	4876	+	208	Signal peptidase I
	7040	7678	-	212	Pyrrolidone-carboxylate peptidase
DP	171541	172920	+	459	Dipeptidase
	6502	7920	-	472	Putative Xaa-His dipeptidase
	59440	60528	+	362	Proline dipeptidase
	45802	47226	+	474	Dipeptidase

Abbreviations: **AA** amino acid; **AP** aminoprotease; **PR** protease; **EP** endopeptidase; **P** peptidase; **DP** dipeptidase

B *Enterococcus faecium* ZGBP5-52

Product type	Start	End	Strand	AA Lenght	Product
AP	24562	25746	-	394	Isoaspartyl aminopeptidase @ Asp-X dipeptidase
	47290	48369	+	359	Glutamyl aminopeptidase; Deblocking aminopeptidase
	14744	15976	-	410	Tripeptide aminopeptidase
	42365	43693	-	442	Aminopeptidase C
	1380	2612	-	410	Aminopeptidase S (Leu, Val, Phe, Tyr preference)
	4211	4975	+	254	Methionine aminopeptidase
	23468	24532	+	354	Aminopeptidase YpdF (MP-, MA-, MS-, AP-, NP- specific)
	3856	4938	+	360	Putative aminopeptidase YsdC
PR	126872	127744	+	290	Membrane protease family protein BA0301
	61225	61902	+	225	Intracellular protease
	20452	21738	-	428	Uncharacterized zinc protease YmfH
	21731	22999	-	422	FIG001621: Zinc protease
	76522	77208	+	228	Late competence protein ComC, processing protease
	33588	34286	+	232	FIG056164: rhomboid family serine protease
	14688	15395	-	235	Putative membrane protease YugP
	38786	40294	+	502	ATP-dependent Zn protease
	67492	68115	+	207	SOS-response repressor and protease LexA
	21499	23733	-	744	ATP-dependent Clp protease, ATP-binding subunit ClpE
	29875	30423	+	182	ATP-dependent protease subunit HslV
	30437	31837	+	466	ATP-dependent hsl protease ATP-binding subunit HslU
	50169	51458	-	429	Serine protease, DegP/HtrA, do-like
	48690	49343	-	217	ClpCP protease substrate adapter protein MecA
	17323	17916	+	197	ATP-dependent Clp protease proteolytic subunit ClpP
	34172	35197	+	341	Protease IV
	16368	17411	+	347	Lon-like protease with PDZ domain
	10149	10262	+	37	CAAX amino terminal protease family protein
	18737	19987	-	416	ATP-dependent Clp protease ATP-binding subunit ClpX
	4560	5810	+	416	Uncharacterized protease YrrO
	915	3407	+	830	ATP-dependent Clp protease, ATP-binding subunit ClpC
	5556	5675	-	39	Uncharacterized protein sporadically distributed in bacteria and archaea, not a Lon-type protease
EP	14156	16063	-	635	Neutral endopeptidase O
P	119944	122289	-	781	Multimodular transpeptidase-transglycosylase
	24562	25746	-	394	Isoaspartyl aminopeptidase @ Asp-X dipeptidase
	29412	29876	-	154	Lipoprotein signal peptidase
	53185	55566	+	793	Multimodular transpeptidase-transglycosylase/Penicillin-binding protein 1A/1B (PBP1)
	20585	21136	+	183	Signal peptidase I
	56859	57503	+	214	Pyrrolidone-carboxylate peptidase
	54170	54739	+	189	Signal peptidase I
	9668	10186	+	172	Signal peptidase type I
	3628	4551	+	307	Peptidase, U32 family small subunit [C1]
	4684	6855	+	723	Multimodular transpeptidase-transglycosylase
DP	117285	118391	-	368	Proline dipeptidase
	24562	25746	-	394	Isoaspartyl aminopeptidase @ Asp-X dipeptidase
	50379	51719	+	446	Putative Xaa-His dipeptidase
	44095	45513	+	472	Putative Xaa-His dipeptidase

Abbreviations: **AA** amino acid; **AP** aminoprotease; **PR** protease; **EP** endopeptidase; **P** peptidase; **DP** dipeptidase

C *Enterococcus faecalis* ZGBP5-53

Product type	Start	End	Strand	AA Lenght	Product
AP	84606	85835	+	409	Tripeptide aminopeptidase
	102016	102261	+	81	D-aminopeptidase dipeptide-binding protein DppA
	102261	102863	+	200	D-aminopeptidase dipeptide-binding protein DppA
	81702	82784	+	360	Putative aminopeptidase YsdC
	65601	66665	-	354	Aminopeptidase YpdF (MP-, MA-, MS-, AP-, NP- specific)
	34841	36169	+	442	Aminopeptidase C
	47325	48545	-	406	Tripeptide aminopeptidase
	7738	8814	-	358	Glutamyl aminopeptidase; Deblocking aminopeptidase
	24933	25697	-	254	Methionine aminopeptidase
	27843	29081	+	412	Aminopeptidase S (Leu, Val, Phe, Tyr preference)
PR	215457	216077	-	206	SOS-response repressor and protease LexA
	284743	286146	-	467	ATP-dependent hsl protease ATP-binding subunit HslU
	286161	286709	-	182	ATP-dependent protease subunit HslV
	317600	319033	-	477	Carboxyl-terminal protease
	420770	421636	-	288	Membrane protease family protein BA0301
	469497	470750	-	417	ATP-dependent Clp protease ATP-binding subunit ClpX
	148925	149587	+	220	ClpCP protease substrate adapter protein MecA
	42349	43059	+	236	FIG056164: rhomboid family serine protease
	164027	164707	-	226	Intracellular protease
	134818	135510	+	230	Late competence protein ComC, processing protease
	64327	65631	-	434	Uncharacterized zinc protease YmfH
	65624	66892	-	422	FIG001621: Zinc protease
	2778	5018	+	746	ATP-dependent Clp protease, ATP-binding subunit ClpE
	42294	43346	-	350	Lon-like protease with PDZ domain
	3327	3920	+	197	ATP-dependent Clp protease proteolytic subunit ClpP
	4633	5931	+	432	Serine protease, DegP/HtrA, do-like
	50550	51218	-	222	Membrane-bound protease, CAAX family
	32738	33442	-	234	Putative membrane protease YugP
	14698	15945	-	415	Uncharacterized protease YrrO
	18242	20737	-	831	ATP-dependent Clp protease, ATP-binding subunit ClpC
P	317027	317581	-	184	Signal peptidase I
	360181	360666	-	161	Lipoprotein signal peptidase
	373994	376363	+	789	Multimodular transpeptidase-transglycosylase
	100916	102019	+	367	Peptidase, M42 family
	228782	229957	+	391	Peptidase, M20/M25/M40 family
	188769	189494	-	241	Signal peptidase I
	153784	155082	-	432	Peptidase, M20/M25/M40 family
	162587	164923	+	778	Multimodular transpeptidase-transglycosylase/Penicillin-binding protein 1A/1B (PBP1)
	202997	203539	-	180	Signal peptidase I
	80737	81378	+	213	Pyrrolidone-carboxylate peptidase
	29851	32037	-	728	Multimodular transpeptidase-transglycosylase
	67342	68190	+	282	Putative peptidase A
	36082	36618	+	178	Signal peptidase I
	15973	16893	-	306	Peptidase, U32 family small subunit [C1]
DP	379739	380842	+	367	Proline dipeptidase
	47622	49037	-	471	Putative Xaa-His dipeptidase

Abbreviations: AA amino acid; **AP** aminoprotease; **PR** protease; **P** peptidase; **DP** dipeptidase
